# A statistical model for describing and simulating microbial community profiles

**DOI:** 10.1371/journal.pcbi.1008913

**Published:** 2021-09-13

**Authors:** Siyuan Ma, Boyu Ren, Himel Mallick, Yo Sup Moon, Emma Schwager, Sagun Maharjan, Timothy L. Tickle, Yiren Lu, Rachel N. Carmody, Eric A. Franzosa, Lucas Janson, Curtis Huttenhower

**Affiliations:** 1 Harvard Chan Microbiome in Public Health Center, Harvard T.H. Chan School of Public Health, Boston, Massachusetts, United States of America; 2 Department of Biostatistics, Harvard T.H. Chan School of Public Health, Boston, Massachusetts, United States of America; 3 Broad Institute, Cambridge, Massachusetts, United States of America; 4 Department of Human Evolutionary Biology, Harvard University, Cambridge, Massachusetts, United States of America; 5 Department of Statistics, Harvard University, Cambridge, Massachusetts, United States of America; 6 Department of Immunology and Infectious Diseases, Harvard T.H. Chan School of Public Health, Boston, Massachusetts, United States of America; Rutgers University, UNITED STATES

## Abstract

Many methods have been developed for statistical analysis of microbial community profiles, but due to the complex nature of typical microbiome measurements (e.g. sparsity, zero-inflation, non-independence, and compositionality) and of the associated underlying biology, it is difficult to compare or evaluate such methods within a single systematic framework. To address this challenge, we developed SparseDOSSA (Sparse Data Observations for the Simulation of Synthetic Abundances): a statistical model of microbial ecological population structure, which can be used to parameterize real-world microbial community profiles and to simulate new, realistic profiles of known structure for methods evaluation. Specifically, SparseDOSSA’s model captures marginal microbial feature abundances as a zero-inflated log-normal distribution, with additional model components for absolute cell counts and the sequence read generation process, microbe-microbe, and microbe-environment interactions. Together, these allow fully known covariance structure between synthetic features (i.e. “taxa”) or between features and “phenotypes” to be simulated for method benchmarking. Here, we demonstrate SparseDOSSA’s performance for 1) accurately modeling human-associated microbial population profiles; 2) generating synthetic communities with controlled population and ecological structures; 3) spiking-in true positive synthetic associations to benchmark analysis methods; and 4) recapitulating an end-to-end mouse microbiome feeding experiment. Together, these represent the most common analysis types in assessment of real microbial community environmental and epidemiological statistics, thus demonstrating SparseDOSSA’s utility as a general-purpose aid for modeling communities and evaluating quantitative methods. An open-source implementation is available at http://huttenhower.sph.harvard.edu/sparsedossa2.

## Author summary

Researchers have linked the human microbiome (collection of microbes residing on or within human tissues) with a wide range of health and disease conditions, establishing these microorganisms as a vital component of our well-being. However, studies on the microbiome require careful technical considerations, as invalid approaches can (and have been reported to) cause under-detections or false discoveries. To this end, a mathematical model can be used both to describe microbiomes, and to simulate how computational tools might behave for them. For example, researchers could test analysis approaches on simulated microbiomes, thus informing designs and method deployment for real-world studies. This has not previously been possible, due to multiple technical challenges with microbiome data; these are a) often constrained to sum up to a constant (“compositional"), b) enriched for zero measurements (zero-inflated), and c) composed of many, potentially interacting microbes (high-dimensional). We present a statistical model aimed at describing and simulating microbiome datasets, with components targeting all these issues, as well as the accompanying computational algorithm and implementation. The freely available implementation, named SparseDOSSA, is validated through extensive simulation and real-world examinations, and will hopefully be of broad theoretical and practical use to researchers in microbiome epidemiology and microbial ecology.

This is a *PLOS Computational Biology* Methods paper.

## Introduction

Microbial community research has increasingly benefited from study designs inspired by molecular epidemiology, particularly with the goal of associating features of the human microbiome with health and disease [1]. This has enabled discoveries ranging from overall ecological dysbiosis in gut community structure during inflammatory bowel disease (IBD) [2] to specific microbial species, strains, and gene families linked to colorectal cancer (CRC) [3]. However, in almost all cases, existing statistical methods for genetic, transcriptional, metabolomic, or other molecular epidemiology cannot be accurately applied directly to microbiome measurements, due to their unique measurement error, noise, zero-inflation, compositional, and non-independence properties [4,5]. This has led to inaccuracy issues in the literature, such as confounding, uncorrected population structure, batch effects, and a high rate of false positives [6–9]. There is thus an unmet need for statistical frameworks capable of capturing all aspects of microbiome epidemiology, both for the sake of accurately parameterizing and testing real community profiles, and for “reversing” parameterized models to simulate controlled, synthetic microbiomes for accurate methodology evaluation.

Transcriptional biomarker discovery has a similar history, in which early statistics to associate gene expression patterns with human phenotypes were met with challenges of noise, dimensionality, and test appropriateness [10]. This led to some of the first models for gene expression integrating features of underlying transcriptional biology, different assay platforms, and measurement noise [11]. These were in turn also “reversed” to provide simulated expression data for methods evaluation under guaranteed, controlled circumstances [12], permitting some of the first truly quantitative transcriptional epidemiology and comparative methods evaluation [13].

Models of microbial community structure are similarly important, and both their biological structure and measurement technologies are quite distinct from those for other sources of short-read sequence generation [14]. Microbial community profiles can be derived roughly equivalently from either amplicon (e.g. 16S rRNA gene) or metagenomic shotgun sequencing, and they consist of the (typically compositional) counts or proportions of taxa, genes, pathways, or other features derived from the source sequencing data. Like other types of molecular epidemiology profiles, they are typically a) high-dimensional (number of features equivalent to or surpassing sample size) [1] and b) require both feature-feature and sample-sample biological interactions (i.e. correlations or population structure) to be accounted for [15].

Additionally, microbiome data possess further unique properties that prohibit direct application of models from other molecular epidemiology research. They are considerably more sparse, i.e. zero-inflated, both due to low sequencing depth and biological absence [1]. As a result, in different settings, either biological presence/absence of microbial features or their abundances can be linked to phenotypes [16]. Microbial abundances from sequencing are also near-universally available only on a relative (compositional) scale, thus constrained to sum up to a constant. The combination of general high-dimensional statistical challenges with those unique to ecological profiles have impeded the development of a single, universal model of microbial feature structure.

As such, most previous strategies for modeling or simulating microbial community profiles (typically for methods evaluation) have been relatively simple [5]. Here, we will use “features” and “profiles” to refer to the quantification of taxa or other entities (e.g. genes or pathways) as counts or relative abundances from microbial community sequencing. McMurdie and Holmes [5] adopted deterministic mixing and multinomial sampling for simulating microbial taxa count observations; it thus does not allow for interaction between microbial features, nor does it model biological (as opposed to technical) absences. Similarly, Thorsen *et al*. [17] adopted random resampling of real-world data for simulating “new” microbial features and samples, indirectly violating compositionality and, again, excluding possible feature-feature interactions. In recent works, metaSPARSim [18] adopted a formal statistical model specifically for simulation of 16S rRNA gene amplicon-sequenced microbial observations (here abbreviated 16S), namely, the gamma-multivariate hypergeometric (gamma-MHG) distribution. However, the gamma-MHG model, itself an over-dispersed version of the multinomial model, still does not allow for biological absences or feature-feature interactions. Additionally, the model’s sampling implementation requires iteration over read depth for a given sample, which induces impractically high computation burdens to achieve realistic sequencing depths [1]. Prost *et al*. [19] proposed a zero-inflated multivariate Gaussian copula model (see **Methods** section **Identifiability**), but for the different goal of microbial feature interaction inference and with a substantially different optimization process. None of these frameworks formally capture microbial covariation with real or simulated covariates. In addition to other uses of such models, this is perhaps the most important aspect needed for benchmarking applications, where it enables estimation of power, false discovery rates, and effect sizes for microbiome epidemiology.

To address these gaps, we present SparseDOSSA (Sparse Data Observations for the Simulation of Synthetic Abundance), a statistical model that can be used to capture and, in turn, simulate realistic microbial community profiles. Motivated by the biological and technical data generation mechanisms and properties of microbial abundance observations, SparseDOSSA has model layers for a) zero-inflated marginal microbial abundances, b) penalized estimation of high-dimensional feature-feature interactions, c) enforced normalization to address compositionality, and d) spiking-in of controlled microbe-microbe and microbe-environment covariation for benchmarking. We demonstrate through validations that the current implementation version, SparseDOSSA 2, accurately captures microbial community population and ecological structures across different environments, host phenotypes, and sequencing technologies, and is capable of recapitulating comparable, realistic synthetic profiles (note that we subsequently include the version number only when indicating an implementation-version-specific feature). We also show example applications in microbiome study design power analysis and in recapitulating a complex end-to-end mouse microbiome feeding experiment. An open-source implementation of and documentation for SparseDOSSA 2 are available through R/Bioconductor and at http://huttenhower.sph.harvard.edu/sparsedossa2.

## Results

### A statistical model for microbial community profiles

**Fig 1:**
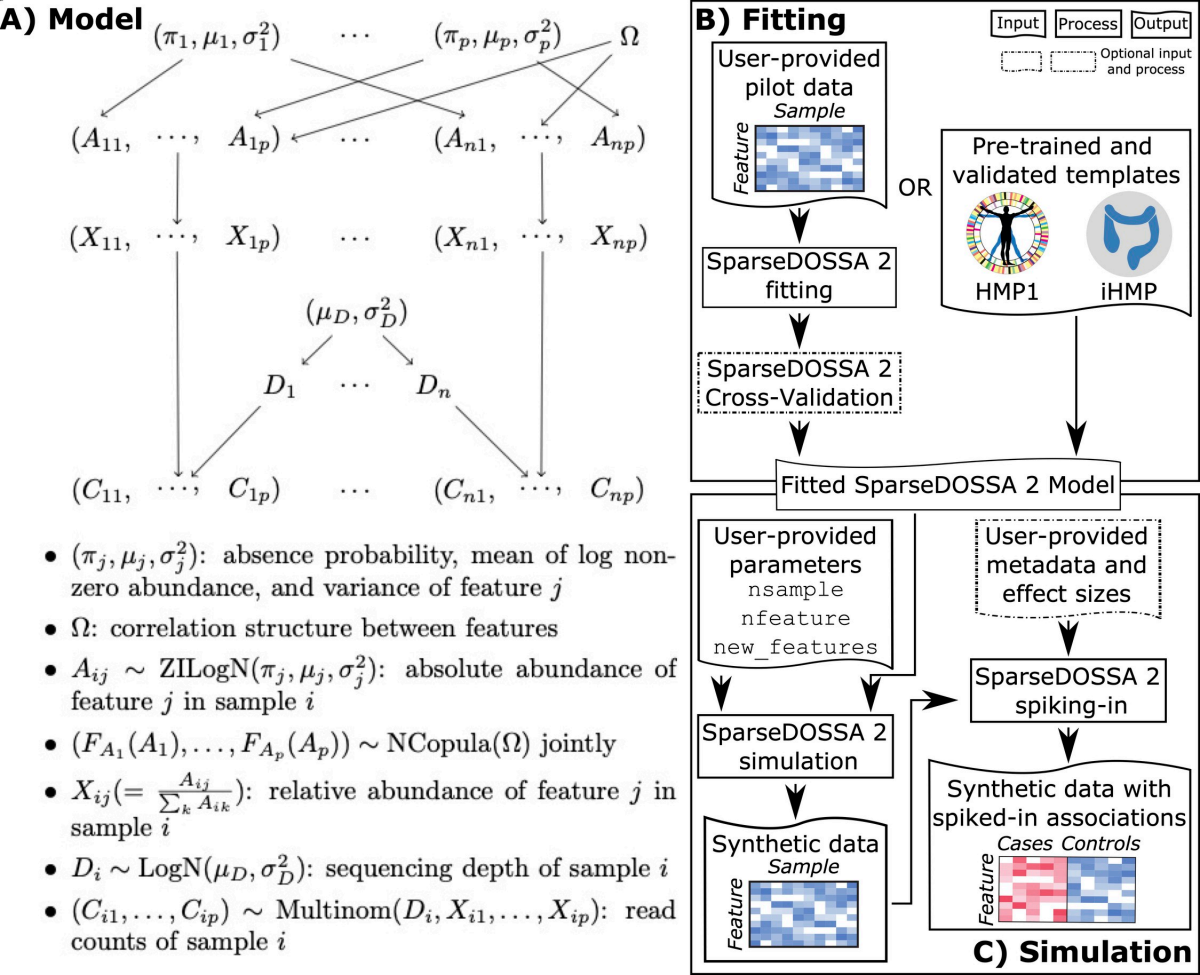
A hierarchical model for microbial community feature profiles. **A)** SparseDOSSA comprises a hierarchical model to capture the generation mechanism of microbial sequencing counts, including components for “hidden” absolute abundances, sequencing depth (and thus compositional relative abundances), zero inflation, and feature-feature and feature-environment interactions. Notations not defined in the figure: $F_{A_{j}}(\cdot )$: cumulative density function (CDF) for the absolute abundance of feature *A*_*j*_. *μ*_*D*_, $\sigma_{D}^{2}$: mean and variance of the log normal sequencing depth distribution. **B)** SparseDOSSA can be fitted to varied microbial community types using cross-validation procedures by users; the software also provides pre-trained models are provided for human microbiome template datasets. This allows for **C)** simulation of either null or "true positive" association spiked-in synthetic datasets, to facilitate microbiome benchmarking or power analysis studies.

SparseDOSSA is a hierarchical model for microbial count and relative abundance profiles (**Fig 1**), with components specifically accommodating the major distributional characteristics of such data, namely zero-inflation, compositionality (and thus sequencing depth), feature-feature non-independence, feature-environment interactions, and high-dimensionality. Briefly (**Fig 1A**, details in **Methods**), the model a) specifies zero-inflated log-normal marginal distributions for each microbial feature to allow for both biological and technical absences, b) imposes distributions on the “absolute”, i.e. pre-normalized, microbial abundances to satisfy compositionality (similar to models such as the Dirichlet [20] or gamma-MGH [18]), c) models feature-feature correlations through a multivariate Gaussian copula [21], and d) adopts a penalized fitting procedure to address high-dimensionality [22]. Conditional on feature relative abundances and total read depth, count observations are modeled with a standard multinomial sampling procedure, and per-sample read depth is modelled with a log-normal distribution. For implementation, we adopted a penalized Expectation-Maximization procedure for model fitting, and we have evaluated and provided options for cross-validated selection of the optimal penalization parameter (**Methods**). SparseDOSSA 2 is implemented as an R/Bioconductor package (http://huttenhower.sph.harvard.edu/sparsedossa2). Note that this differs somewhat from our previous implementation of SparseDOSSA that was already publicly available; we provide a summary of changes between the versions in **Methods**. SparseDOSSA can be accurately fit to a wide variety of different microbial community structures to capture both (inferred) absolute and relative count observations (**Fig 1B and 1C**, details below).

### SparseDOSSA accurately recapitulates real-world microbial community structures

**Fig 2:**
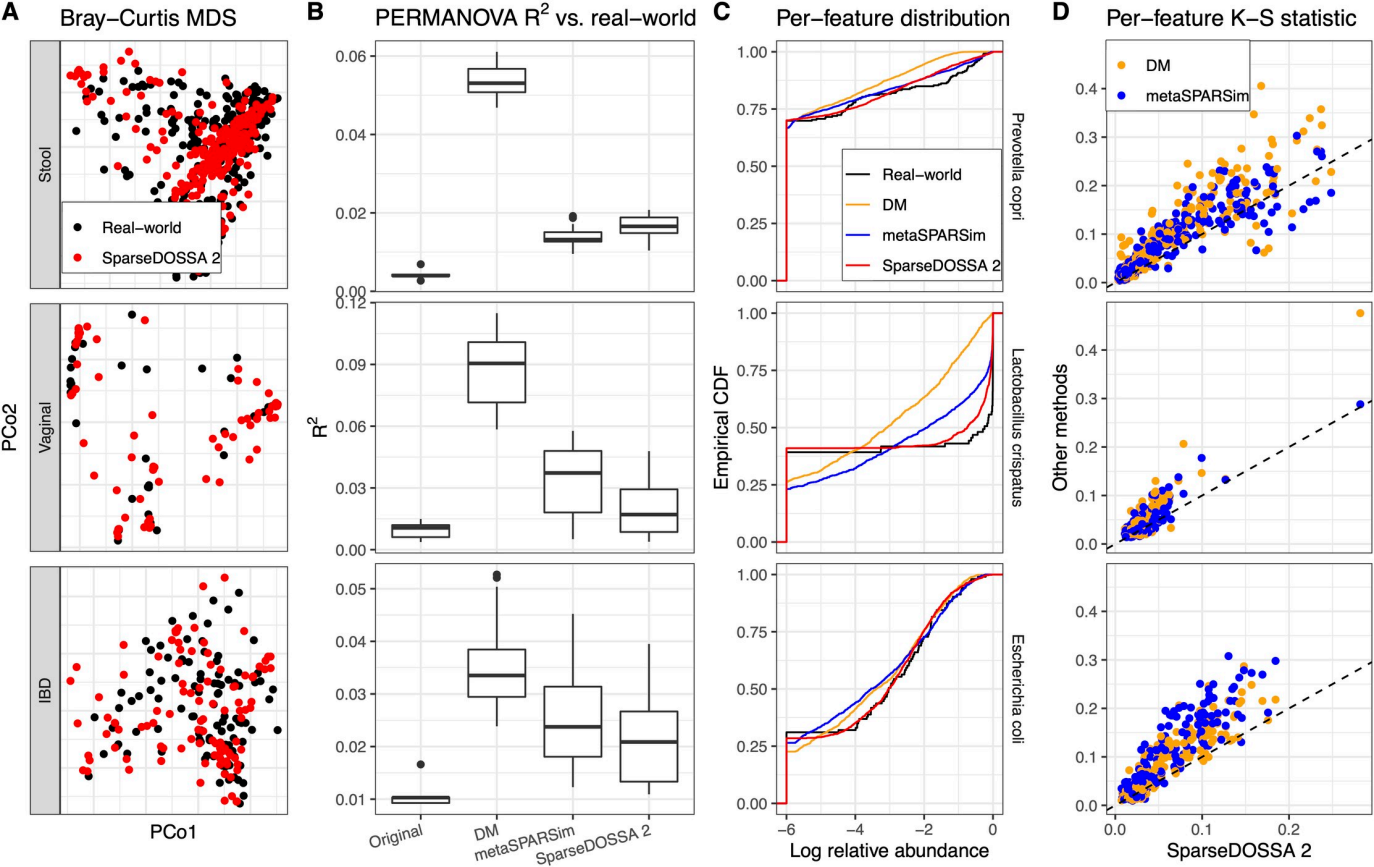
SparseDOSSA accurately recapitulates different microbial community structures. We compared SparseDOSSA 2 simulated microbial counts versus those of three human microbiome training template datasets (Stool, Vaginal, and IBD). **A)** Bray-Curtis ordination shows global agreement between SparseDOSSA simulated microbial abundance profiles and those of their originating real-world populations. **B)** This was quantified by PERMANOVA *R*^2^ statistics, showing that SparseDOSSA simulated samples were significantly less systematically differentiated from their targets than existing DM and metaSPARSim methods in almost all cases (Wilcoxon rank sum test p-values included in **S3 Table**). *R*^2^ compared against randomly split original real-world data are included as baseline controls. **C)** Representative features from each environment are similarly distributed between real-world and SparseDOSSA simulated samples, as shown in empirical cumulative distribution functions (CDFs) of log-10 relative abundances (with pseudo value 1e-6 to visually represent zeros). **D)** Per-feature Kolmogorov-Smirnov summary statistics quantify that SparseDOSSA outperforms existing methods in simulating realistic feature-level relative abundance distributions. First, the similarity between the model-simulated feature abundance distribution versus that in the real-world dataset is quantified with K-S statistics. Then, the K-S statistics for SparseDOSSA and the other two models (DM and metaSPARSim) are plotted on the x- and y-axis, respectively (each point representing one feature, smaller K-S statistics represent better approximation). Lastly, the K-S statistics of SparseDOSSA versus other models are formally tested using Wilcoxon signed rank tests (p-values are significant and included in **S4 Table**).

We validated SparseDOSSA’s ability to accurately capture realistic microbial community feature profiles by quantifying its performance across a variety of real-world datasets (**Fig 2 and S1 and S2 Tables**). The studies used include: 1,2) taxonomic profiles from shotgun sequenced metagenomes of healthy human stool and posterior fornix samples from the HMP1-II, hereafter referred to as “Stool” and “Vaginal” [23], 3) shotgun sequenced stool metagenomes of inflammatory bowel disease (IBD) patients from the HMP2 Inflammatory Bowel Disease Multi-omics Database (IBDMDB, abbreviated as “IBD”) [2], and 4) 16S rRNA gene sequenced murine distal gut communities after diet perturbation [24]. By evaluating the model in different cohorts, we established its robustness under different community phenotypes, habitats (i.e. body sites), overall ecological structures, and sequencing technologies (**S1 Table**).

SparseDOSSA 2 captured community parameters and re-simulated microbial profiles with overall community structures that accurately reflected those of the original, real-world ecologies, better than alternative methods (**Fig 2A and 2B**), across all human datasets (murine study results reported in separate section). Overall, simulated communities yielded the same patterns of global beta-diversity as were contained within each modeled dataset (**Fig 2A**). This was quantitatively compared against alternative models (Dirichlet-multinomial, DM [20] and gamma-MGH, namely metaSPARSim [18]) with the PERMANOVA *R*^2^ statistic [25] (**Methods**). We calculated ecological Bray-Curtis dissimilarities between real-world microbial profiles and those simulated by each evaluated method. We then quantified the total variability in the combined dissimilarities that could be attributed to real-world versus simulation difference, expressed as the PERMANOVA *R*^2^. Smaller *R*^2^s thus indicate less deviation of the simulated community structures from the real-world target and better performance of the model.

Across almost all evaluated community types, SparseDOSSA 2 generated significantly smaller *R*^2^ statistics over 25 simulation iterations than existing methods (Wilcoxon rank sum tests p < 0.05), indicating better fit to and recapitulation of the targeted communities (**Fig 2B,** testing results in **S3 Table**). Notably, this was consistent in both the human gut (Stool, IBD), where community structure forms continuous “gradients” of microbial composition [9], and the human vaginal environment (Vaginal), where communities are often characterized by a few discrete types with dominant species [26]. Only for the Stool dataset did SparseDOSSA slightly underperform when compared to metaSPARSim in terms of *R*^2^ statistic, while still outperforming with respect to per-feature distributions (**Fig 2D**). Additionally, metaSPARSim’s simulation procedure can take as much as ~10x longer than SparseDOSSA 2 (**S1 Fig**), which is prohibitive for realistic data sizes (especially for benchmarking or power-analysis efforts requiring multiple simulations per parameter configuration, or for Monte-Carlo calculations). Based on the above comparative performance results, we conclude that, when evaluated for overall community structures, SparseDOSSA can capture microbial feature profiles that closely resemble those of real-world microbiomes.

The SparseDOSSA model also provided the best recapitulations of individual features’ relative abundances (**Fig 2C and 2D**). For representative features in each environment, the empirical cumulative distribution function (CDF) curves of samples show that SparseDOSSA simulated abundances closely resemble those of the real-world data (**Fig 2C**). Quantitatively, for each set of microbial features, we measured the difference of distributions between re-simulated and real-world (modeled) relative abundances with the Kolmogorov-Smirnov summary statistic (K-S, see **Methods**). The resulting K-S summaries provides a distance between the distribution of each feature’s relative abundances across simulated vs. modeled real-world communities. Smaller K-S statistics thus indicate better performance of the model. SparseDOSSA better approximated the targeted real-world per-feature distributions than existing methods across all evaluated datasets (**Fig 2D**), reaching statistical significance in each case (**S4 Table,** Wilcoxon signed rank tests p < 0.05). In addition to simulating existing microbial features, SparseDOSSA 2 also provides the functionality to simulate new features that resemble the targeted environment’s ecological characteristics (**Methods**) and was validated to generate “Stool-like”, “Vaginal-like”, or “IBD-like” new features in terms of prevalence, abundance, and variability for each of the tested datasets (**S2 Fig**). Thus both in overall community structure modeling and in per-feature models, SparseDOSSA was able to accurately capture and re-simulate realistic microbial observations better than alternative approaches.

### SparseDOSSA captures covariation among microbes and with real or simulated “phenotypes”

Once the SparseDOSSA model is fit to a real-world microbial community profile, the “reversed” version of the model can be used not only to simulate similar, controlled ecologies, but to introduce artificial, known feature-feature or feature-covariate associations to the simulated profiles (i.e. metadata “spike-ins”). This is implemented by first capturing the “null” state of targeted real-world studies as described above and by subsequently modifying the fit model parameters to induce artificial associations. Compared to existing spiking-in paradigms [5,17,18], the model includes two important improvements (**Methods**). First, SparseDOSSA can model a wide variety of covariates—discrete, continuous, or any combination thereof—with multivariable linear modelling, and can thus accommodate simulations of realistic microbiome population study designs with multiple phenotypes, exposures, or confounders [1]. Second, associations with both non-zero (abundance) and zero-inflated (prevalence) components of microbial features can be captured, along with clearly defined effect sizes (fold change or odds ratio, see **Methods**). This enables rigorous evaluations of, for example, differential abundance testing methods for their statistical performance (e.g. power or false positive rates).

**Fig 3:**
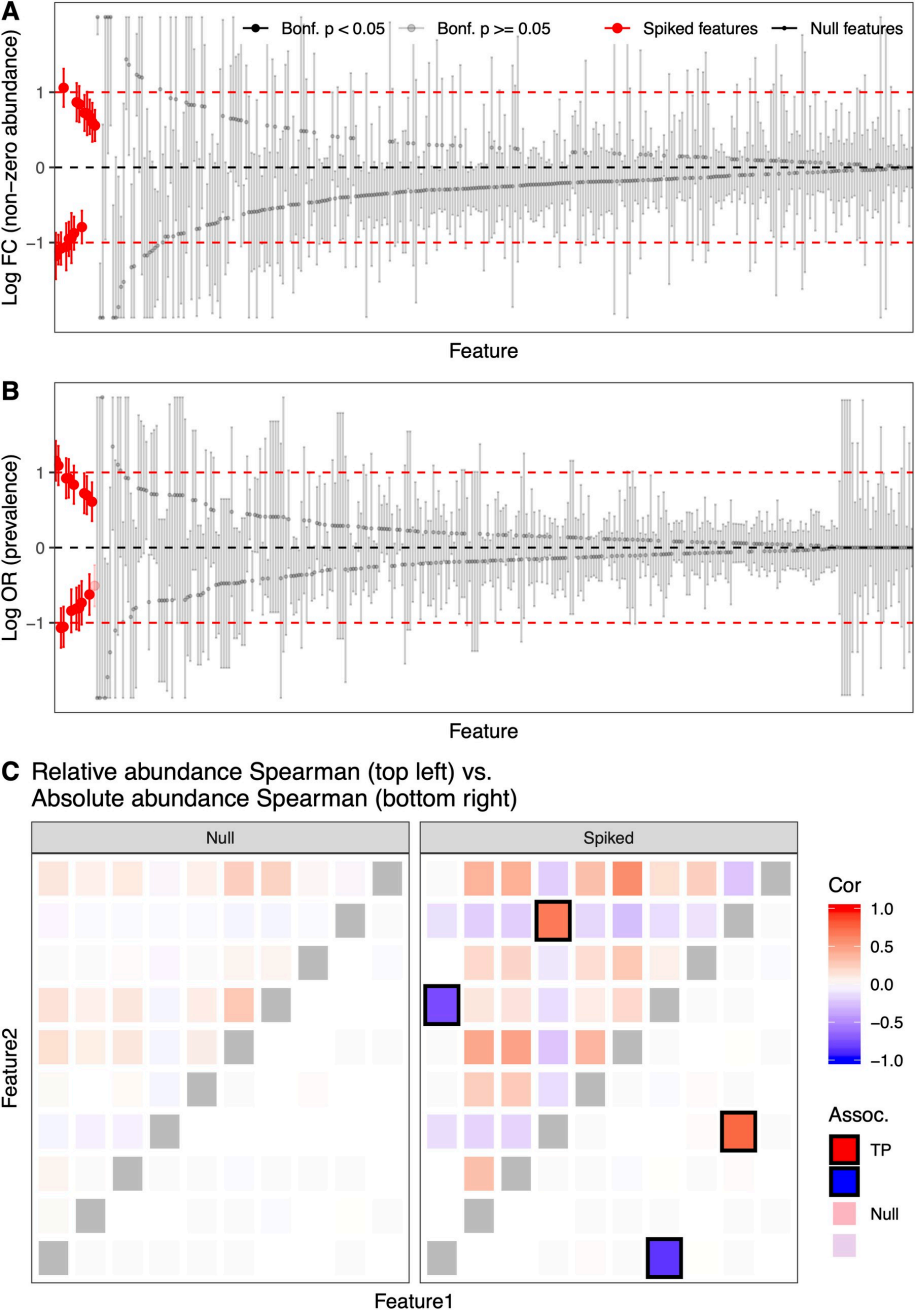
SparseDOSSA can add feature-phenotype and feature-feature associations to modeled microbial community simulations. **A,B)** SparseDOSSA 2 correctly simulated feature-phenotype associations targeting the prescribed non-zero relative abundance (**A**) and prevalence (**B**) effect sizes of the spiked features, while maintaining non-associations of null features. True associated (spiked) microbial features (red) are well differentiated from null features (black), through Bonferroni corrected p-values (non-significant features marked in gray; test based on linear/generalized linear regression against the spiked metadata variable, see **Methods** for details). The horizontal dashed lines indicate true spike-in effect sizes: red lines for the positive and negative true effect sizes, respectively, and the black line for null effect (0). **C**) SparseDOSSA can also prescribe feature-feature associations. Bottom right triangles are Spearman correlations in the simulated absolute abundances. As prescribed, only true association feature pairs are correlated. Top right triangles are Spearman correlations in the corresponding, simulated relative abundances. Note that in this example, Spearman correlation does not differentiate between true (“biological”) covariations versus those induced spuriously due to compositionality (as is also the case in the underlying data on which SparseDOSSA’s model is fit). As expected, both true signals and spurious correlations caused by compositionality can be observed for such data. TP: true positives.

Based on models fit to the Stool and Vaginal communities, SparseDOSSA 2 accurately introduced, or “spiked-in” associations for control phenotypes in a new, simulated population (**Figs 3 and S3**). Specifically, for the Stool dataset, we introduced a binary covariate (similar to e.g. a case / control contrast) with non-zero effects on 16 (5% of the total 332) microbial features’ abundances (**Fig 3A**) and prevalences (**Fig 3B).** Features were selected to ensure the highest effective sample size (**Methods**). For simulated associations of the “phenotype” with feature abundances, log fold change of non-zero relative abundances largely agreed with the target effect sizes within 95% confidence levels (**Fig 3A**). Prevalence log odds ratios were also as targeted (**Fig 3B**), with effects in relative abundances mostly agreeing with the prescribed effect sizes. Similar abundance and prevalence results were consistently reproduced in the Vaginal environment (**S3 Fig**).

In addition to modeling associations between microbial features and external covariates, SparseDOSSA can also model community ecological interactions (i.e. correlations between microbial features, or feature-feature “spike-ins”, **Figs 3C and S4**). First, we note that SparseDOSSA simulated microbial communities naturally recapitulate important synergies within modeled environments, such as Firmicutes-Bacteroidetes gradients in the human gut and co-exclusion of dominant Lactobacillus species in the vaginal microbiome (**S5 Fig**). For introducing artificial correlation among microbial features, we extended the feature-covariate spiking process above to synthetically associate multiple features with the same hidden covariate (**Methods**). First, a null model fit to the Stool/Vaginal communities contains no true feature-feature associations, only those that manifest spuriously due to compositionality. Starting with this, we modified the model to induce increasingly large feature-feature “ecological” interactions. SparseDOSSA 2 produced both only and exactly the expected true feature-feature associations among absolute abundance components, and the correct induced compositional correlations after simulating the sequencing assay process (**Fig 3C** for subset of Stool results; full Stool/Vaginal results in **S4 Fig**). These results support SparseDOSSA’s ability to modify baseline, null community structures by the introduction of interactions among features or with controlled covariates, which together enable the evaluation of a wide range of statistical approaches to microbiome analysis [15,27,28].

### Modeling environment-specific benchmarking and power estimation

**Fig 4:**
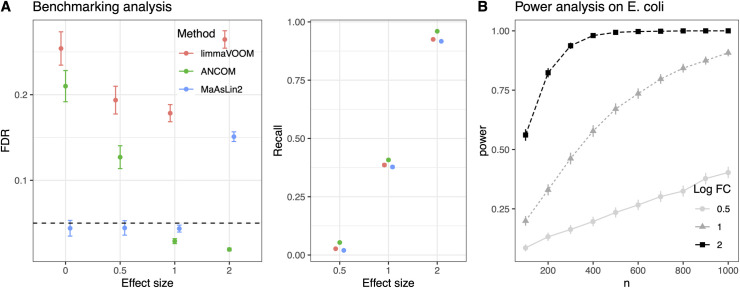
SparseDOSSA enables comparative benchmarking and power analysis of microbial community statistical association tests. For any originating community type of interest, datasets simulated based on a SparseDOSSA model fit can be spiked with known "phenotypes" and feature effect sizes to estimate methods performance (power, FPR, etc.) during (**A**) benchmarking as well as (**B**) power analysis, across controlled combinations of potential effect sizes and sample sizes. Points indicate average performance across simulation repetitions and error bars indicate standard error (**Methods**).

Since most microbiome analysis methods make simplifying assumptions that may or may not be suited to particular ecologies, SparseDOSSA’s flexible model enables power and accuracy estimation in a habitat-specific manner (**Fig 4**). Specifically, by spiking only a limited set of known feature-phenotype associations into an otherwise guaranteed-null model, differential abundance methods can be compared directly to each other in a controlled setting (more details in [29]), enabling targeted method benchmarking (**Fig 4A**) or power analysis (**Fig 4B**).

To demonstrate SparseDOSSA’s use for benchmark comparison of microbial community statistical tests, we again simulated synthetic datasets based on the Stool profiles with "phenotypic" associations spiked-in for 5% of features at varying effect sizes (as in **Fig 3A**). Multiple replicates of the same parameter set were performed to provide performance metric mean and standard errors (**Methods**). Using the resulting gold standards, the performances of three different association tests–limmaVOOM [30], ANCOM [31], and MaAsLin 2 [29]—were similar for power, but false discovery rates varied strikingly (**Fig 4A**). Notably, the MaAsLin 2 generalized linear model showed good FDR control at small to moderate effect sizes. At higher effect sizes, non spiked-in (“null”) features are also called by MaAsLin 2 as differentially abundant. Interestingly, this is because SparseDOSSA’s spike-in effects are imposed on features’ simulated absolute abundances (**Methods**), and high effect size spike-ins thus also induce relative abundance change in null features due to compositionality. This highlights the important difference between true differential abundance effects corresponding to microbes’ biological variation, versus changes post normalization that are driven by other features.

In contrast, ANCOM [31] was designed to account for compositionality and draw inference about hidden absolute abundances; it successfully and maintained FDR under moderate to strong effect sizes. Arguably as a result, however, its performance suffered for small to null effects, presumably because in such cases it is difficult to distinguish between “driver” microbial features with true absolute effects versus those with changes in their relative abundances due to compositionality. Lastly, limmaVoom [30], designed primarily for RNA-Seq data, had inflated FDRs across all cases.

To demonstrate SparseDOSSA’s use for power analysis during microbial community study design, we focused targeted simulation datasets with spiked-in effects on a feature modeled on *Escherichia coli*, as a microbe commonly associated with dysbiosis in the human gut [2]. Using this approach, SparseDOSSA 2 can be used to estimate each association method’s expected power for similar biomarkers and populations. In this example, MaAsLin 2 has high power to detect a two-fold abundance change in "*E*. *coli*" for a sample size of at least ~500 individuals, but greatly reduced power for smaller fold-changes (**Fig 4B**). Since model power for differential abundance testing in sparse, compositional data is extremely difficult to determine parametrically, SparseDOSSA thus provides a way to do so by simulation tailored to any community type or feature of interest, and we provide basic guidelines for this in **Methods**.

### SparseDOSSA reproduces an end-to-end diet-microbiome analysis

**Fig 5:**
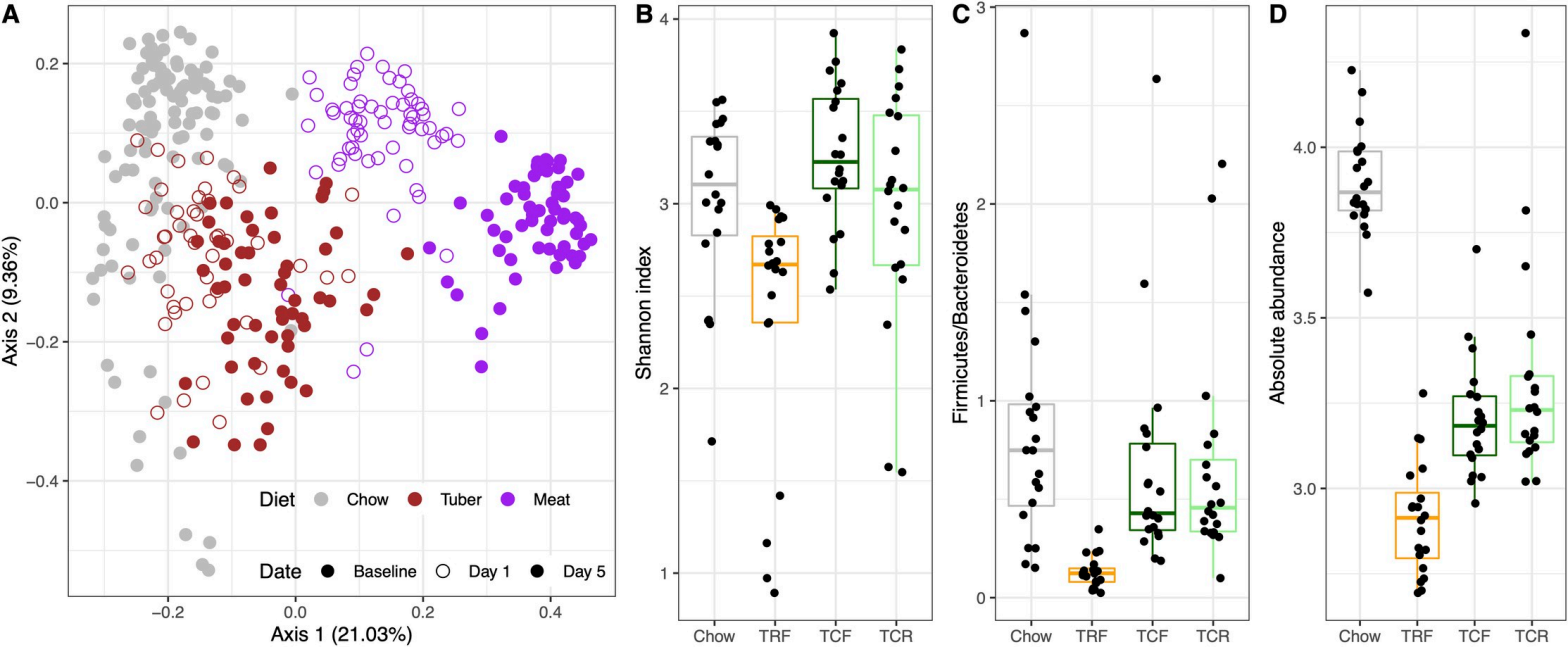
SparseDOSSA correctly models the effects of diet and time on the murine gut microbiome by reproducing effects from amplicon sequencing profiles. **A)** SparseDOSSA 2 was fitted to subsets of samples from [24] that included up to three time points each from collections of mice fed chow, raw or cooked tubers, and meat. The resulting models were then used to simulate controlled microbial community profiles, which correctly reproduced the beta-diversity structures present in the original study (MDS ordination by Bray-Curtis dissimilarities, corresponding to Fig 1A of [24]). The SparseDOSSA model was also able to model and synthetically replicate changes in "Bacteroidetes" and "Firmicutes" phyla in response to raw vs. cooked diets, including **B)** overall community alpha-diversity (Shannon index), **C)** the resulting "Firmicutes" vs. "Bacteroidetes" ratio, and **D)** overall whole-community effective biomass. These correspond to [24]’s Fig 1F–1H, respectively. TRF = raw tuber (free-fd); TCF = cooked tuber (free-fed); TCR = cooked tuber (restricted ration).

In many cases, SparseDOSSA thus captures the properties of microbial community ecologies well enough to reproduce surprisingly specific aspects of their membership and distributions, which we next demonstrated by reproducing an end-to-end example from a longitudinal, interventional diet study investigating the effects of diet on the murine gut microbiome [24] (**Fig 5**). Carmody *et al*. [24] used 16S rRNA gene sequencing to profile changes in the mouse distal gut microbiome under different dietary treatments (chow, raw and cooked tuber, and meat). To determine whether SparseDOSSA could accurately model the microbes, phenotypes, and associations observed over time in these settings, we fitted model parameters for each sample type at different time points and under different treatment assignments (**Methods**).

After re-simulating communities based on these models fits, ordinations of SparseDOSSA results closely mimic the originally observed clustering structure of dietary effects, and even the longitudinal effects of time under treatment (**Fig 5A**, corresponding to Fig 1A of [24]). For quantitative differential abundance effects, based on the observed difference of raw diet (TRF) samples when compared against cooked/free-fed (TCF) and cooked/restricted (TCR) within the tuber diet group [24], we additionally applied SparseDOSSA 2’s spike-in procedure to simulate a ~2x fold increase in the abundance of Bacteroidetes OTUs in TCF/TCR when compared to TCF samples, and a ~2x fold decrease in Firmicutes OTUs (**Methods**). Consequently, the simulated samples displayed similar differential outcomes in community diversity as measured by Shannon index, as well as Firmicutes/Bacteroidetes ratios, as seen in the original study (**Fig 5B and 5C**, corresponding Fig 1F–1G of [24]).

Interestingly, even though per-feature absolute abundances are theoretically unidentifiable in the SparseDOSSA model (**Methods**), we note the spiking-procedure recapitulated the decreased total cell counts in TRF (**Fig 5D**, corresponding to Fig 1H of [24]) that [24] also observed via quantitative PCR. The difference between chow versus tuber diets, on the other hand, is completely attributable to SparseDOSSA’s framework, as one can arbitrarily modify the average absolute abundances of our fit to the chow or tuber diet samples, but still yield exactly the same relative abundance profiles. These real-world application results highlight SparseDOSSA’s adaptability to community phenotypes and treatment effects, as well as demonstrate its performance for amplicon sequence datasets and microbial communities associated with non-human hosts.

## Discussion

Here, we have developed a statistical model, implemented in the R package SparseDOSSA 2, for fitting and/or simulating microbial community profiles. These can comprise taxonomic abundances (i.e. relative abundances or counts) from shotgun metagenomic or amplicon sequencing; although not evaluated here, the model is in principle also appropriate for other microbial feature abundances (e.g. genes or pathways). The model can be fit to communities with different host-associated or environmental ecological structures, and it accurately captures their fundamental characteristics, including the distribution of abundances across community members and the diversities of microbial composition across populations. In addition, to support quantitative benchmarking of new methods for microbial community statistics and epidemiology, SparseDOSSA is able to reliably induce user-specified correlation structures involving feature-covariate or feature-feature associations in simulated ecologies. This was demonstrated not only *in silico*, but by end-to-end reproduction of results paralleling those in an interventional mouse feeding study. The underlying generative model thus efficiently and effectively summarizes real microbial communities and recapitulates their latent structure in a manner that is both computationally efficient and statistically principled.

The SparseDOSSA model assumes that the characteristics of a template (real) microbial community are well-captured by the distributions it includes for each component (individual features, feature-feature relationships, sparsity, etc.) More specifically, this requires that 1) the non-zero component of absolute abundances is approximately log-normal, 2) that feature-feature association structure is sparse (as captured by the penalized estimation procedure), and 3) that intrinsic population substructure among samples are absent in the template dataset (i.e. before SparseDOSSA 2 itself optionally spikes-in any such structure). The last assumption 4) that sequencing depths within study are themselves log-normal typically has minimal impact on model fitting or usage. The third assumption holds reasonably well even when any correlation structure originally present is weak or rare relative to overall microbial variance, or affects only a small proportion of features, similar to the assumption of “few differential transcripts” used in most RNA-seq models [32]. Second, inasmuch as the read count of each feature depends on its own observed mean, variance, and sparsity, SparseDOSSA 2’s simulated data will replicate the marginal distribution of the originating template community. This guarantee on the null distribution of subsequently generated communities allows correlation structure (with samples or among features) to be optionally added in isolation for evaluation of microbial community analysis methods. The first assumption is most approximate—it is generally true for ecologically diverse communities, which empirically follow power-law or log-normal behaviors (with a few abundant organisms and a long tail representing the increasingly rare biosphere). However, as discussed above, its violation leads to small residual systematic biases (<0.5%) in communities where tails of rare organisms are more truncated than expected.

Perhaps the greatest strength of the model is its application in simulating microbial community profiles, which we have emphasized and validated here. Most previous methods for associating microbial features with covariates [31,33–35] or with each other [15,28,36,37] have relied on heterogeneous, one-off models not necessarily reflective of any one “real” microbial community type, or of the diversity of ecological configurations observed in the wild (e.g. the human gut vs. vaginal microbiome vs. soil). By providing a model that can accurately capture many different community types, remove any existing structure through null distributions, and re-introduce known, controlled structure (microbial or covariate), we hope to provide a convenient, unified framework with which statistical methods can be validated specifically for their environments of interest (e.g. human microbiome epidemiology vs. environmental ecological interactions). In addition to this application, while not emphasized here, the model’s parameterization can be used to directly inspect or compare microbial communities. For example, the estimates of absence probabilities π_*j*_ for important microbes *j* of interest in specific human populations (e.g. *Prevotella* in the Westernized vs. non-Westernized gut [38]), or the relationships between π_*j*_ vs. mean log-abundance μ_*j*_ across microbes (i.e. prevalence vs. abundance) are directly informative as to their neutral dispersal vs. selection [39]. To some degree this is evident from the murine feeding example above, but most such applications remain to be demonstrated in broader “real-world” datasets.

Relatedly, SparseDOSSA successfully reproduced reported dietary effects on the mouse gut microbiome [24], without assuming such differences *a priori* (**Fig 5**). By fitting our model on microbial observations of separate treatment groups and time points, we allowed SparseDOSSA to adapt to each subset independently, but without assumptions on the existence or magnitude of differences between them. The emergent reproduction of differentiation by diet in the resulting synthetic communities and features (**Fig 5A**) exemplifies SparseDOSSA’s utility in capturing environment- or treatment-specific dynamics of real-world microbial communities. In parallel, by introducing effects within each dietary group, SparseDOSSA’s per-feature spike-in procedure was able to reproduce structural microbial community changes such as overall diversity and whole-phylum abundance trade-offs. Together, this end-to-end real-world case study highlights SparseDOSSA’s two key functionalities while also testing a non-human, amplicon-sequenced application context: generating realistic microbial community profiles that closely mimic the targeted environment, and introducing covariate spiked-in microbial perturbations to simulate treatment effects.

With respect to this second use case (covariate effects spike-in), existing simulation models often adopt the simplistic approach of modifying the abundances of taxa in the null community to introduce known associations [5,18]. SparseDOSSA, in comparison, utilizes rigorous perturbation models to explicitly specify the marginal means of taxa as functions of chosen covariates. This a) enables much more flexible applications such as the inclusion of confounders or random effects (by incorporating them as covariates), and b) yields spiked-in datasets that are strictly compatible with the standard assumptions of (generalized) linear models. Alternatively, differentiation between simple binary (case-versus-control) contrasts could be achieved with our current model by training SparseDOSSA separately on the two corresponding population subsets, given that each was sufficiently large to serve as a template.

Our modeling and simulation procedure for generating feature-feature correlations is, in turn, directly based off the feature-covariate model and comparatively more restrictive; we expect to explore more rigorous and flexible approaches in future work, since any one “correct” way to model ecological associations in absolute vs. relative abundance space is not clear *a priori* [36]. Another related area for future work is in the specific model used for absolute abundances, which are not well-understood from currently available data; our current assumption holds if the total biomass of “typical” communities does not change under “typical” circumstances, but this is obviously quite qualitative. Direct measurements of microbial biomass in some environments such as the human gut have sometimes shown this within approximately one fold change [40,41], but not in all cases, and certainly not during extreme perturbations such as antibiotics [42].

Thus the SparseDOSSA model simultaneously provides a conceptual framework with which to capture key aspects of microbial ecologies and their members, a simulation system for benchmarking statistical methods that assess correlation structure in microbial community profiles, and a set of marginal parameters for each community and community type of lower dimensionality and potentially reduced noise relative to raw data. The last, while again not yet explored, could allow sample metadata covariates to be more accurately tested for association with microbial features, or tested for association with microbial community features indirectly (e.g. via their prevalence or mean when present). In addition to the areas discussed above, future expansions of the model might include longitudinal structure or other interdependencies among samples (i.e. population substructure), as well as diversifying the application areas for the model (e.g. for power calculations during study design). We note that for introducing correlation among samples with the current implementation (e.g. for simulating longitudinal observations), the user can already create subject-specific “random effect” metadata variables, which then induce tunably non-independent microbial abundances through the existing metadata spike-in procedure. This creates artificial subject-specific effects for the simulated microbial profiles and thus within-subject longitudinal correlations. As currently implemented, SparseDOSSA 2 provides an end-to-end system that enables reproducible and efficient validation of quantitative methods applied to microbial community taxonomic profiles, allowing fair comparisons to be made between different methods or studies to establish a consistent baseline for statistical validation.

## Methods

### The SparseDOSSA model

SparseDOSSA uses the following data generation mechanism to parameterize microbial community profiles: a) environments/samples contain microbes with absolute abundances *A* b) these are normalized to relative abundances *X*, which c) can be measured via sequenced counts *C*. As detailed in **Fig 1A**, our model specification for these components is:

- For the unobserved absolute abundances *A* = (*A*_1_, *A*_2_, …, *A*_*p*_), we specify a Gaussian copula model [21] with zero-inflated log normal marginal distributions. Specifically, this involves assuming hidden multivariate Gaussian variables *g* = (*g*_1_, *g*_2_, …, *g*_*p*_) for the microbial features and a mapping of these variables to the corresponding absolute abundances (*A*_1_, *A*_2_, …, *A*_*p*_):
  ○ *g* ~ *MVN*(0, *Ω*^−1^). That is, each *g*_*j*_ is a standard *N*(0, 1) variable and their correlation matrix is *Ω*^−1^.
  ○ Each *g*_*j*_ is mapped to *A*_*j*_ such that *A*_*j*_ follows a zero-inflated log-normal distribution, parameterized by absence probability (*π*_*j*_) and mean and variability of non-zero log abundances (*μ*_*j*_, *σ*_*j*_^2^):
    ■ *A*_*j*_ = 0 if *g*_*j*_ < *Φ*^−1^(*π*_*j*_)
    ■ $A_{j}={F_{A_{j}}}^{-1}(\Phi (g_{j})|\pi_{j},\mu_{j},{\sigma_{j}}^{2})$ if *g*_*j*_ ≥ *Φ*^−1^(*π*_*j*_)
  Where *Φ* is the standard normal cumulative density function and $F_{A_{j}}$ is the cumulative density function of the zero-inflated log-normal distribution, parameterized by *π*_*j*_, *μ*_*j*_, *σ*_*j*_^2^.

It follows from our model specification that, marginally, *A*_*j*_ follows the prescribed zero-inflated log-normal distribution exactly:

○ With probability *π*_*j*_, *A*_*j*_ = 0
○ With probability 1 − *π*_*j*_, log *A*_*j*_ ~ *N*(*μ*_*j*_, *σ*_*j*_^2^)

Jointly, correlations between microbial features’ absolute abundances are characterized through the copula parameter *Ω*. The benefit of adopting a copula model is to separate the parameterization and estimation of a joint distribution into its marginal and correlation components; this is illustrated in the **model fitting** subsection below.

- Relative abundances are directly normalized from absolute abundances: $X_{j}=\frac{A_{j}}{\sum_{k=1}^{p}A_{k}}$. This by definition satisfies compositionality ($\sum_{j=1}^{p}X_{j}=1$). Also note that because *A*_*j*_’s are zero-inflated, this directly induces zero-inflation (i.e. biological absence) in *X*_*j*_’s.
- For a given sample *i* with sequencing depth *D*_*i*_, its per-feature read counts (*C*_*i*1_, *C*_*i*2_, …, *C*_*ip*_) are assumed to follow a multinomial distribution with individual features’ probabilities given by *X*_*ij*_. That is, (*C*_*i*1_, *C*_*i*2_, …, *C*_*ip*_) ~ *MultiNom*(*D*_*i*_, *X*_*i*1_, *X*_*i*2_, …, *X*_*ip*_), thus also allowing technical zeros.
- Lastly, we assume the sequencing depth *D*_*i*_ across samples follows a log-normal distribution. That is, *D*_*i*_ ~ *LogN(μ*_*D*_, *σ*_*D*_^2^).

### Model likelihood

It is helpful to clarify the likelihood of our model given its parameterization. First, we derive *f*_*A*_, the likelihood for the unobserved absolute abundances *A*. The likelihood of observed data, as we show later, is an integration of *f*_*A*_. For illustration purposes, we first note the special case where *A*_*j*_ are not zero-inflated. That is, *π*_*j*_ = 0 for all *j*’s. In this case, we have that:

$$f_{A}(A_{1},\cdots ,A_{p}|\pi_{1},\mu_{1},{\sigma_{1}}^{2},\cdots ,\pi_{p},\mu_{p},{\sigma_{p}}^{2},\Omega )=f_{g}(g_{1},\cdots ,g_{p}|\Omega )\times \prod_{j}\frac{f_{A_{j}}(A_{j}|\pi_{j},\mu_{j},{\sigma_{j}}^{2})}{\phi (g_{j})}$$

Where *g*_*j*_ is as defined above: $g_{j}=\Phi^{-1}(F_{A_{j}}(A_{j}))$ and *ϕ*(·) is the standard normal density function. The equality follows by noting that the second term (the product) is the Jacobian of the mapping $g\to A:A_{j}={F_{A_{j}}}^{-1}(\Phi (g_{j}))$. When one or more *A*_*j*_’s are zero-inflated, the mapping *g*→*A* is not one-to-one, and the right hand side of the equality requires integration over *g*_*j*_’s that map to zero-valued *A*_*j*_’s:

$$f_{A}(A_{1},\cdots ,A_{p}|\pi_{1},\mu_{1},{\sigma_{1}}^{2},\cdots ,\pi_{p},\mu_{p},{\sigma_{p}}^{2},\Omega )=\int_{g_{j}\leq \Phi^{-1}(1-\pi_{j}),j\in \{j:A_{j}=0\}}f_{g}(g_{1},\cdots ,g_{p}|\Omega )dg\times \prod_{j\in \{j:A_{j}>0\}}\frac{f_{A_{j}}(A_{j}|\pi_{j},\mu_{j},{\sigma_{j}}^{2})}{\phi (g_{j})}$$

To derive the likelihood for relative abundances *X*, we note that *X*, jointly with the total absolute abundance *A*^*Σ*^ (*A*^*Σ*^≔∑_*j*_*A*_*j*_), forms a one-to-one mapping with the absolute abundances *A* (*A* = *A*^*Σ*^*X*). Thus, the density function for *X*, *f*_*X*_, can be obtained through integration of $f_{A^{\Sigma },X}$, which is simply *f*_*A*_ multiplied by the Jacobian of the transformation:

$$f_{X}(X_{1},\cdots ,X_{p}|\pi_{1},\mu_{1},{\sigma_{1}}^{2},\cdots ,\pi_{p},\mu_{p},{\sigma_{p}}^{2},\Omega )=\int f_{A}(A^{\Sigma }X|\pi_{1},\mu_{1},{\sigma_{1}}^{2},\cdots ,\pi_{p},\mu_{p},{\sigma_{p}}^{2},\Omega )\times (A^{\Sigma }{)}^{|\{j:A_{j}>0\}|-1}dA^{\Sigma }$$ (1)

Lastly, for the observed microbial count data *C*, the proper likelihood is:

$$f_{C}(C_{1},\cdots ,C_{p}|\pi_{1},\mu_{1},{\sigma_{1}}^{2},\cdots ,\pi_{p},\mu_{p},{\sigma_{p}}^{2},\Omega )=\int f_{C|X}(C|X)f_{X}(X|\pi_{1},\mu_{1},{\sigma_{1}}^{2},\cdots ,\pi_{p},\mu_{p},{\sigma_{p}}^{2},\Omega )dX$$

Where *f*_*C*|*X*_(*C*) is the multinomial likelihood for microbial counts given their relative abundances. In practice, to simplify computation, during model fitting we replace this likelihood with

$$f_{C}(C_{1},\cdots ,C_{p}|\pi_{1},\mu_{1},{\sigma_{1}}^{2},\cdots ,\pi_{p},\mu_{p},{\sigma_{p}}^{2},\Omega )\approx f_{X}(\hat{X}|\pi_{1},\mu_{1},{\sigma_{1}}^{2},\cdots ,\pi_{p},\mu_{p},{\sigma_{p}}^{2},\Omega )\times const$$ (2)

Where $\hat{X}$ is the multinomial MLE for *X* given observed *C*, i.e., $\hat{X}=\frac{C}{\sum_{j}C_{j}}$, *const* is a normalizing constant not involving the parameters. The approximation is acceptable because with modern sequencing depth [1], *f*_*C*|*X*_(*C*|*X*) (as function of *X*) is highly concentrated around $\hat{X}$. The right-hand side of (2) is what we aim to maximize for estimation of our model’s parameters, *π*_1_, *μ*_1_, *σ*_1_^2^,⋯,*π*_*p*_,*μ*_*p*_,*σ*_*p*_^2^, and *Ω*.

### Identifiability

It is important to note that likelihood (1) is unidentifiable. That is, there exist different values of the parameter set (*π*_1_, *μ*_1_, *σ*_1_^2^,⋯,*π*_*p*_, *μ*_*p*_, *σ*_*p*_^2^, *Ω*) that yield the same likelihood *f*_*X*_ (and consequently *f*_*C*_). Intuitively, this is because *X* are normalized from absolute abundances *A*, and different *A* values can map to the same normalized relative abundances *X*—this is thus typical of any compositional setting. Regarding the identifiability of our parameters, we build on the results of [43], which is a special case of our model where *π*_*j*_ = 0 for all *j*’s. Specifically, we note that:

- *π*_*j*_’s are identifiable, as *X*_*j*_ = 0 ⇔ *A*_*j*_ = 0
- *μ*_1_,⋯,*μ*_*p*_ are identifiable up to a constant. That is, *μ*_1_,⋯,*μ*_*p*_ and *μ*_1_+*c*,⋯,*μ*_*p*_+*c* lead to the same likelihood, for any constant *c*. For this reason, in our model estimation we impose the (arbitrary) constraint that ∑_*j*_*μ*_*j*_ = 0.
- *σ*_1_^2^,⋯,*σ*_*p*_^2^ are identifiable, given *μ*_1_,⋯,*μ*_*p*_ and *Ω*. One can note that when *π*_*j*_ = 1 for all *j*’s, our likelihood degenerates to that in [43] with explicit analytical forms.
- *Ω* is not identifiable. Again, consider the special case that *π*_*j*_ = 1 and *σ*_*j*_ = 1, the form of *f*_*X*_ is explicit and involves $\Omega -\frac{\Omega 1_{p}{1_{p}}^{\prime }\Omega }{{1_{p}}^{\prime }\Omega 1_{p}}$, which is a multiple-to-one mapping from *Ω*. The issue of non-identifiable correlation matrices for microbiome abundance data has been noted and addressed in many previous works; refer to [15,27,28,43] for a partial list. We adopt the technique used in many of these previous works, namely *L*_1_ penalization on *Ω*, to simultaneously address the identifiability issue as well as high-dimensionality for generic estimation of large covariance matrices [22].

We note that, importantly, good performance of SparseDOSSA does not require correct estimation of its non-identifiable parameters. Rather, so long as SparseDOSSA provides a configuration of estimated parameters that yields a joint distribution of relative abundances (*X*_1_,⋯,*X*_*p*_) that closely approximates that of the original data, its application case for capturing or simulating microbial observations is achieved. Thus, our proposed solution for non-identifiability, though not guaranteed to correctly estimate the unknown true parameters, does satisfy typical applications.

### Model fitting

Given our model specification and its (non-)identifiability, we propose to minimize the following penalized negative log-likelihood function for solving the parameter set *Θ* = (*π*_1_, *μ*_1_, *σ*_1_^2^,⋯,*π*_*p*_, *μ*_*p*_, *σ*_*p*_^2^, *Ω*) (the sequencing depth parameters (*μ*_*D*_, *σ*_*D*_^2^) can be fitted independently on per-sample read depths with maximum likelihood):

$$\sum_{i=1}^{n}-log\;f_{X}(\hat{X_{i}}|\pi_{1},\mu_{1},{\sigma_{1}}^{2},\cdots ,\pi_{p},\mu_{p},{\sigma_{p}}^{2},\Omega )+\lambda ||\Omega |{|}_{1}$$

Subject to the constraint for *μ*_*j*_ as specified above: ∑_*j*_*μ*_*j*_ = 0. As such, *λ*>0 is a penalizing tuning parameter, which we choose with K-fold cross-validation in practice (five folds by default). ${\hat{X}}_{i}$ can be either existing relative abundance estimations or, as specified above, normalized from count observations (${\hat{X}}_{ij}=\frac{C_{ij}}{\sum_{j}C_{ij}}$).

As specified in (1), the likelihood function *f*_*X*_ involves integration over *A*^*Σ*^ and is not analytically tractable. Numerically, we propose the following penalized expectation-maximization algorithm [44] for model fitting:

1. Initialize ${\hat{\Theta }}^{\left(0\right)}={{\hat{\pi }}_{1}}^{\left(0\right)},{{\hat{\mu }}_{1}}^{\left(0\right)},{{\hat{\sigma }}_{1}}^{\left(0\right)},\cdots ,{\pi_{p}}^{\left(0\right)},{\mu_{p}}^{\left(0\right)},{\sigma_{p}}^{\left(0\right)},\Omega^{\left(0\right)}$ by fitting a multivariate log-normal distribution on ${\hat{X}}_{i}’s$.
2. During the *r*-th iteration:
  - E-step: calculate expectation $l^{\left(r\right)}(X|\Theta )=\sum_{i}E_{{A^{\Sigma }}_{i}|X_{i};{\hat{\Theta }}^{\left(r-1\right)}}log\;f_{A}({A^{\Sigma }}_{i}X_{i}|\Theta )\times ({A^{\Sigma }}_{i}{)}^{|\{j:A_{ij}>0\}|-1}$.
  - Penalized M-step: maximize −*l*^(*r*)^(*X*|*Θ*)+*λ*||*Ω*||_1_ with respect to *Θ* to obtain ${\hat{\Theta }}^{\left(r\right)}$. Note that ${\hat{\pi }}_{j}’s$ do not require updates. ${{\hat{\mu }}_{j}}^{\left(r\right)}$ and ${{\hat{\sigma }}_{j}}^{\left(r\right)}$ can be solved analytically. ${\hat{\Omega }}^{\left(r\right)}$ can be solved with standard graphical lasso [22].
3. Iterate until convergence.

### Generating synthetic microbial observations and simulating new features

Given that our model is fully parametric, synthetic microbial observations, including (hidden) absolute abundances, normalized relative abundances, and sequencing counts, can be generated following the same specifications as described above. To provide model parameters, the user can adopt one of the pre-trained sets included with the software or use the SparseDOSSA 2 training procedure to estimate parameters from any microbial template dataset suited for their simulation case.

Users may also be interested in generating “new” microbial features from the same ecological environment. For this, SparseDOSSA additionally models the per-feature parameters (*π*_*j*_, *μ*_*j*_, *σ*_*j*_^2^) with a three-dimensional non-parametric distribution *F*. That is, across features, (*π*_*j*_, *μ*_*j*_, *σ*_*j*_^2^)~*F*. Given a set of SparseDOSSA fitted results $\left({\hat{\pi }}_{1},{\hat{\mu }}_{1},{\hat{\sigma }}_{1}^{2},\ldots ,{\hat{\pi }}_{p},{\hat{\mu }}_{p},{\hat{\sigma }}_{p}^{2}\right)$, *F* can be estimated with a three-dimensional normal kernel density estimator [45]. The estimated $\hat{F}$ can then be used to simulate new microbial features that follow the ecological characteristics (i.e., prevalence, abundance, and variability) of the fitted environment (**S2 Fig**).

### Association spike-in

SparseDOSSA adopts linear and generalized linear models for flexible spiking-in in both microbial features’ non-zero abundances and prevalences, based on covariates. Let *Z*_*i*_ be the vector of covariate(s) for sample *i* and *β* be the targeted corresponding effect sizes (coefficients). To spike in associations between feature *j*’s abundance and covariates *Z*_*i*_, we modify the feature’s non-zero mean log absolute abundance parameter *μ*_*j*_ across samples. Specifically, the post spike-in mean log abundance is modified as

$$\mu_{ij}={\mu_{j}+Z_{i}}^{\prime }\beta_{abundance}$$

For the *i*-th sample, *A*_*ij*_ can be generated with *ZILogN*(*π*_*j*_, *μ*_*ij*_, *σ*_*j*_^2^) instead of the original *ZILogN*(*π*_*j*_, *μ*_*j*_, *σ*_*j*_^2^). This dictates that *A*_*ij*_’s are associated with *Z*_*i*_ in their mean non-zero log abundances. As *μ*_*ij*_ are specified on the log scale, *β*_*abundance*_, by definition, corresponds to log fold changes.

The prevalence spike-in similarly is specified via the logistic model; we modify the presence probability parameter (1−*π*_*j*_) across samples:

$$log(\frac{1-\pi_{ij}}{\pi_{ij}})=log(\frac{1-\pi_{j}}{\pi_{j}})+{Z_{i}}^{\prime }\beta_{prevalence}$$

And generate *A*_*ij*_’s correspondingly. This introduces an association between the covariates *Z*_*i*_ and feature *j*’s prevalence, with *β*_*prevalence*_ corresponding to log odds ratios of the feature being present. The multivariate linear modelling approach for specifying the association effects for both abundance and prevalence allows us to flexibly incorporate different variable types (e.g. binary, continuous, etc.) and study designs (e.g. existence of confounders).

We note that, importantly, our spiking-in procedure is performed on the absolute abundances, *A*, which induces differential effects in relative abundances *X* (**Fig 3A and 3B**). The main benefit of this approach is that both the spiked-in microbial features and the “null” (i.e. non-spiked features) are clearly defined. The alternative—specifying effects for *X*_*j*_—is conceptually difficult. As *X* is compositional (sums to 1), prescribing enrichment effects (higher abundance or prevalence) for some microbial features must by definition lead to depletion effects for certain other features. This renders it difficult to clearly define and separate the set of "true positive" spiked-in microbial features and the set of null features. SparseDOSSA’s definition of effects for absolute abundances in its spike-in procedure align with recent efforts to rigorously characterize microbial differential abundance effects under the constraint of compositionality [31,46]. Empirically, we note that prescribed log fold changes or odds ratios for *A*_*j*_ often lead to similar effect sizes in the relative abundances *X*_*j*_ for the spiked-in feature *j*’s (**Fig 3A and 3B**).

Lastly, we note that the spiking-in procedure with metadata variables can be used to simulate association effects between pairs of microbial features (**Fig 3C**). Specifically, we first simulate a hidden covariate *Z* with standard normal distribution. For a pair of features *j*_1_, and *j*_2_, to enforce positive correlations between the two absolute abundances $A_{j_{1}}$ and $A_{j_{2}}$, we simulate for them to be associated with *Z* in the same direction:

$$\mu_{ij_{1}}=\mu_{j_{1}}+Z_{i}\beta_{abundance}$$

$$\mu_{ij_{2}}=\mu_{j_{2}}+Z_{i}\beta_{abundance}$$

$$log(\frac{1-\pi_{ij_{1}}}{\pi_{ij_{1}}})=log(\frac{1-\pi_{j_{1}}}{\pi_{j_{1}}})+Z_{i}\beta_{prevalence}$$

$$log(\frac{1-\pi_{ij_{2}}}{\pi_{ij_{2}}})=log(\frac{1-\pi_{j_{2}}}{\pi_{j_{2}}})+Z_{i}\beta_{prevalence}$$

Where *β*_*abundance*_ = *β*_*prevalence*_ = *β* can be viewed here as the effect size specifying the strength of correlation between $A_{j_{1}}$ and $A_{j_{2}}$. To spike in negative correlation between the two, we simply keep *β* as the effect for one of the features and use −*β* for the other.

### Comparison between SparseDOSSA and SparseDOSSA 2

A previous implementation of SparseDOSSA has also been publicly available [47]. To help the reader understand the difference between SparseDOSSA’s old and new versions, we list the major updates below:

1. In the new version, zero-inflated log-normal modelling of microbial feature abundances is imposed on absolute abundances *A*_*ij*_, instead of directly on the relative abundances *X*_*ij*_, to better satisfy compositional constraints of such data types.
2. Microbial feature-feature correlations are now explicitly modelled with the Gaussian copula component; this was not present in the previous version.
3. For simulating new microbial features, which requires modelling of the triplet (*π*_*j*_, *μ*_*j*_, *σ*_*j*_^2^), the previous implementation adopted a log-normal distribution on *μ*_*j*_, with corresponding linear and logit link functions for *σ*_*j*_ and *π*_*j*_. This is relaxed in the new version with the three-dimensional nonparametric Gaussian kernel density estimator to allow for additional flexibilities.
4. For spiking-in of association microbial features and metadata variables, the previous version only allows for abundance association spike-in. It also specifies effect sizes through a non-linear transformation that can be difficult for the user to interpret. The new version expanded this functionality by allowing both abundance and presence spiking-in; it also specifies effect sizes directly through linear and generalized linear models (log fold change/odds ratio), allowing more straightforward interpretations.
5. The necessary algorithm and implementation changes for the aforementioned model updates, including our new EM algorithm for model fitting, updated and expanded real-world templates for direct simulation, and software interface updates.

### Evaluation with real-world datasets

For evaluation and comparison of microbiome simulation methods, we examined three real-world datasets with different host environments and disease statuses (**S1 Table**) [2,23]. We subset publicly available species level profiles from [23] (all healthy) to baseline time point stool (Stool) and posterior fornix (Vaginal) samples, and those from [2] to baseline time point IBD samples (IBD, including Crohn’s disease and ulcerative colitis patients). We additionally removed samples with lower than ~3,000 reads mapped to identified taxa and features present in less than 3 samples. The datasets’ dimensions (sample size, number of features), post processing and filtering, are included in **S1 Table**. A summary of all simulation evaluations performed in this work is included in **S2 Table**. Evaluation scripts used are publicly available at https://github.com/biobakery/sparsedossa_paper.

To evaluate the performance of individual methods, we randomly partitioned each dataset (Stool, Vaginal, IBD) into halves for five iterations. For each partitioning, we fit the parameterization/simulation methods (DM, metaSPARSim, and SparseDOSSA 2) on one half of the data (training). We then simulated synthetic microbial observations with the same sample size using the fitted results. Lastly, we compared the synthetic observations with the other half of the partitioned data (testing) in terms of both overall dissimilarity with PERMANOVA [25] and per-feature distribution differences (methods detailed below). Within each partitioning, this simulation was also performed five times for each method. That is, each method was used to randomly simulate five synthetic datasets for comparison with the testing half. The partitioning procedure allows us to evaluate method performance without a model overfitting effect. The DM was fitted using R package “dirmult” and metaSPARSim was fitted using the implementation referred to in its publication [18]. For metaSPARSim fitting, the percent not zero filter for features was set to 0 instead of the default 0.2. In our evaluation this led to an observed performance increase (thus a favorable assessment), likely due to the existence of many highly zero inflated microbial species.

To evaluate overall dissimilarity between the original and synthetic samples, for each partitioning we combined the testing half of the original samples with the simulated datasets (five for each fitted method). We calculated the sample-to-sample Bray-Curtis dissimilarity matrix *D* on the combined dataset. The univariate PERMANOVA model, *D*~*I*{*Sample is simulated*} was fitted. The corresponding *R*^2^ statistic quantifies the percentage of variability between samples attributable to the difference between original "real-world" as compared to simulated samples. Smaller *R*^2^ statistics indicate less difference, and better performance of the simulation method. For each method, a total of 25 PERMANOVA evaluations (5 original dataset partitioning × 5 simulation) was performed for each real-world dataset. Lastly, we additionally evaluated the *R*^2^ between the training and testing halves of a dataset for each partitioning; this yields an estimation of minimum achievable *R*^2^’s for each dataset. *R*^2^’s between methods are compared with Wilcoxon rank sum tests (**S3 Table**).

To evaluate the difference between distributions of individual features in the original and synthetic datasets, we simply combined the synthetic datasets generated across all partitioning and simulation repetitions. An individual feature was compared for its relative abundance distribution between the original real-world data and combined synthetic samples. This was quantified with the Kolmogorov-Smirnov (K-S) summary statistic, which is defined as the largest absolute difference between the empirical cumulative distribution functions of the real-world and synthetic abundances. Smaller K-S statistics indicate better approximation of the targeted real-world distributions with the simulation method. To compare overall per-feature performance between models, the paired K-S summary statistics between SparseDOSSA 2 and alternative models (DM and metaSPARSim) were compared using Wilcoxon signed rank tests (matched by features, **S4 Table**).

### Association spike-in evaluation

We simulated spiked-in associations between microbial features and a synthetic case/control variable, based on the SparseDOSSA 2 fitted results. A total of 1,000 synthetic samples were simulated (500 cases and 500 controls). For non-zero abundance spike-in (**Fig 3A**), the top 5% (16 total) most prevalent features were selected for spiking-in; this yields the highest effective sample size for the selected features because our abundance spiking-in targets only the non-zero component of a feature’s distribution. Half of the features were spiked for a targeted log fold change (*β*_*abundance*_) of 1 in cases compared to controls, and the other half were spiked for a log fold change of -1. Actual log fold changes in the simulated relative abundances, along with 95% confidence intervals, were calculated by performing a linear regression on the log transformed non-zero relative abundances for each feature.

Similarly, for prevalence spiking-in (**Fig 3B**), the top 5% features with prevalence closest to 0.5 were selected; as with abundance spike-ins, this was to ensure the spiked-in features had the highest effective sample size, as the association between a binary outcome (presence/absence here) and a binary covariate (case/control) is best-powered when the sample distribution is balanced across all different outcome/covariate combinations [48]. Again, half of these features were spiked for a targeted log odds ratio (*β*_*prevalence*_) of 1 in cases compared to controls, and the other half were spiked for a log odds ratio of -1. Actual log odds ratios of the simulated feature prevalence, along with 95% confidence intervals, were calculated by performing a logistic regression on the presence for each feature.

For simulation of feature-feature associations, we first set the correlation between feature pairs in SparseDOSSA 2 to zero (i.e., *Ω* = *I* where *I* is the identity matrix). This ensures that feature absolute abundances are independent in the “null” dataset (**Fig 3C** left panel, bottom right), whereas spurious correlation still exists in relative abundances due to compositionality. Two random pairs (four features) in the top ten most abundant features were selected for non-zero feature-feature association spike-in. As specified in **Methods** above, we simulated two independent normal synthetic hidden metadata variables, one for each feature pair to be associated. For the first feature pair, they were spiked in both abundance and prevalence with the same effect (*β* = *β*_*abundance*_ = *β*_*prevalence*_) at varying sizes, for positive association. The second pair were spiked with opposing effects (*β* for one, −*β* for the other) for negative association. We used Spearman correlation to estimate the empirical association between feature pairs in the simulated absolute and relative abundances. Target association effect size was also varied (*β* of 0, 1, 2, and 5) to showcase the relative signals of “true” associations that exist for both absolute and relative abundances, and spurious associations that are only induced in relative abundances due to compositionality (**Figs 3C and S4**).

### Benchmarking

Since “true” associations with prescribed effect sizes are known for SparseDOSSA synthetic datasets, they can be used for benchmarking microbiome analysis methods as well as for power analysis of microbiome study designs. For benchmarking analysis (**Fig 4A**), we again selected the top 5% (16 total) most prevalent features in the Stool dataset to perform abundance spike-in, such that the selected features had the highest effective sample size. A total of 200 microbial profiles were simulated to be associated with a balanced binary metadata (100 cases, 100 controls). We varied effect sizes with half spiked features at *β*_*abundance*_ = (0, 0.5, 1.2) and the other half with *β*_*abundance*_ = (0, −0.5, −1, −2), correspondingly (in the effect size 0 case no spike-in was performed and microbial profiles are generated independently of metadata). A total of 500 random simulations were performed for each parameter combination. We applied existing differential abundance analysis methods to detect the spiked-in features in each simulation dataset [29–31], with individual method configurations as reported in our previous benchmarking analysis [29]. We summarized the empirical power and FDR of a method in one simulation dataset, across the twenty random replicates for each parameter configuration, and reported the mean and standard error in **Fig 4A**.

### Power analysis

For showcasing SparseDOSSA’s utility in a power analysis, we spiked in non-zero abundance associations with a balanced case-control variable for a simulated species parameterized by fitting *Escherichia coli*. This was performed at varying effect sizes (log fold change, *β*_*abundance*_ = (0.5, 1, 2)) and sample sizes (100 to 1000). For each parameter configuration, a total of 500 replicates were simulated. The empirical power and its standard error of using MaAsLin 2 to detect the differential abundant effect in "*E*. *coli*" was summarized across the 500 replicates and reported in **Fig 4B**; this was repeated for each effect size/sample size configuration.

While it remains to build a more formal power analysis using these models, we recommend the following guidelines if using SparseDOSSA directly:

- Based on the planned study design, if a pilot data set is available, the fitting functionality can be used to estimate the parameters that would generate similar data sets. Otherwise, researchers can adopt one of the pre-fitted templates that best approximate their target environment.
- Researchers can then simulate many datasets (e.g. hundreds or thousands) with the fitted parameters, across a range of spike-in effect sizes and sample sizes. We note that the SparseDOSSA 2 effect size has straightforward quantitative interpretations: log fold changes for abundance spike-in, and log odds-ratios for prevalence spike-in (details on model in **Association spike-in** subsection of **Methods**).
- This should be followed by conducting the hypothesis testing that is planned for the study on each of the simulated datasets. This allows for a measure of how often the “true” (spiked-in) associations are detected for a given sample size (i.e. statistical power). Additionally, the researcher can investigate whether other methods of testing can deliver comparable results to simulation-based estimates from SparseDOSSA 2.

### Murine diet microbiome analysis

We applied SparseDOSSA 2 to the longitudinal diet dataset of the mouse gut microbiome in [24], to show that our method is capable of reproducing a complex study’s findings. To recapitulate the longitudinal diet effect as reported in [24]’s Fig 1A, we fitted SparseDOSSA 2 separately on 1) the control Chow diet samples at baseline, 2,3) Tuber diet samples at day 1 and day 5, separately, and 4,5) Meat diet samples at day 1 and day 5, separately. This approach allows SparseDOSSA 2 to independently fit subsets of the data, without assuming *a priori* the observed differences noted in [24]. We then used SparseDOSSA 2 fitted results to simulate synthetic observations for each diet/timepoint combination, with five times the original sample size (to reduce variability due to random sampling). Bray-Curtis MDS ordination on these synthetic data displayed a striking resemblance to that observed in [24] (**Fig 5A**), in that a) communities cluster according to dietary treatment, and 2) this response is consistent after one day of switching from chow to whole-food diets and is strengthened at day 5.

We next reproduced the differential gut microbial profiles observed in mice fed raw versus tuber diets as presented in [24]’s Fig 1F–1G. [24] adopted three different types of Tuber diet: the raw/free-fed (TRF), the cooked/free-fed (TCF), and the cooked/restricted (TCR). This study presented that on the phylum level, TRF induced enrichment of Bacteroidetes and depletion of Firmicutes when compared to TCF/TCR. We applied SparseDOSSA 2’s feature spike-in procedure to approximate this effect. Specifically, we generated a balanced, three category (TRF/TCF/TCR) variable. Based on our fitted model of the Tuber diet at day 5, we spiked in a two-fold (*β*_*abundance*_ = log 2) increase in the non-zero abundance of Bacteroidetes OTUs and a two-fold decrease in Firmicutes OTUs (*β*_*abundance*_ = −log 2) in TRF samples when compared to TCF/TCR samples. This roughly agrees with the presented results in [24] Fig 1D. We next simulated SparseDOSSA synthetic datasets for both the baseline Chow diet samples (sample size = 20), and the spiked-in Tuber diet samples (60 samples total, 20 each for TRF/TCF/TCR). We calculated the Shannon index and Firmicutes/Bacteroidetes ratios of these samples, and show in **Fig 5B** that they agreed with the corresponding findings presented in [24] Fig 1F–1G.

## Supporting information

S1 Fig Computation time comparison between SparseDOSSA 2 and metaSPARSim, and SparseDOSSA 2’s fitting and simulation processes. All computation evaluated as run on single Intel "Cascade Lake" cores. **A)** SparseDOSSA 2 simulation is faster than metaSPARSim across the evaluated real-world datasets. Results were aggregated across the 25 simulation evaluations (5 original dataset partitionings × 5 simulations) for each dataset. Note that here the simulation sample size n are halved compared to the actual sample size per dataset because only half the samples were simulated according to the training-testing partitioning paradigm (**Methods**). **B)** SparseDOSSA 2 fitting requires significant computation costs up front compared to its simulation process, analogous to sequence search database indexing. These are evaluated across the grid of tuning parameter λs per real-world dataset (fitting and simulation performed for full datasets). We note that the fitting costs can be potentially alleviated by 1) the algorithm is parallelizable and implemented as such, and 2) for a target template the fitting needs to be performed only once, whereas numerous simulation repetitions are required for e.g. benchmarking or power analysis.
(PDF)

S2 Fig SparseDOSSA 2 simulated features have characteristics parameters distributed similarly to those of the real-world targets. In each case, “new” microbial features were simulated with SparseDOSA 2’s three-dimensional Gaussian kernels, and compared with the original features by examining the distribution of their absence probability *π*_*j*_ (logit transformed), mean log non-zero abundance *μ*_*j*_, and standard deviation of log non-zero abundance *σ*_*j*_.
(PDF)

S3 Fig SparseDOSSA 2’s spike-in procedure introduced targeted per-feature differential abundance and prevalence effects in the Vaginal dataset. (PDF)

S4 Fig SparseDOSSA 2’s feature-feature correlation spike-in can induce covariation (as measured by Spearman correlation) of feature absolute abundances at different targeted effect sizes, and consequentially correlation in relative abundances while confounded by compositionality. Top row: stool community results; bottom row: vaginal community results.
(PDF)

S5 Fig SparseDOSSA simulated microbial profiles preserve important feature covariations in the human gut and vaginal environments. We simulated 100,000 microbial abundance profiles each based on the SparseDOSSA Stool and Vaginal models and examined the resulting simulated feature-feature Spearman correlations. Note that Spearman correlation does not differentiate between feature-feature correlations induced by biological interaction and compositionality; it is used purely to characterize co-variation patterns in the simulated microbial profiles, as opposed to making biological claims as to whether they are biological vs. technical. **A)** Simulated Spearman correlations in the top five most abundant Firmicutes-derived features (bottom left, abundance increasing top to bottom), and the top five most abundant Bacteroidetes-derived features (top right, abundance increasing bottom to top) from the Stool SparseDOSSA model. Strong positive correlations were observed among the Firmicutes species, whereas negative ones can often be observed between Firmicutes and Bacteroidetes species, as expected [9]. **B)** Simulated Spearman correlations in the top eight most prevalent and abundant species’ models from the Vaginal SparseDOSSA fit (bottom to top, left to right decreasing in average abundance). We observe strong co-exclusions between dominant Lactobacillus features, and positive correlations among a few less dominant species indicative of joint presence in the absence of Lactobacilli.
(PDF)

S1 Table Sample characteristics of the three real-world studies used for evaluating SparseDOSSA 2’s performance. (XLSX)

S2 Table Summary of all simulation evaluation analyses performed in the manuscript. (XLSX)

S3 Table Wilcoxon rank sum test p-values for the difference of *R*^2^ statistics comparing SparseDOSSA 2 against the two existing methods (corresponding to Fig 2B). (CSV)

S4 Table Wilcoxon signed rank test p-values for the difference of Kolmogorov-Smirnoff statistics comparing SparseDOSSA 2 against the two existing methods (corresponding to Fig 2D). (CSV)
