# Supplementary figures and images for: A statistical model for describing and simulating microbial community profiles
Source: PLoS Comput Biol. 2021 Sep 13;17(9):e1008913. doi: 10.1371/journal.pcbi.1008913 (PMC8491899; doi:10.1371/journal.pcbi.1008913)

## A Simulation time costs

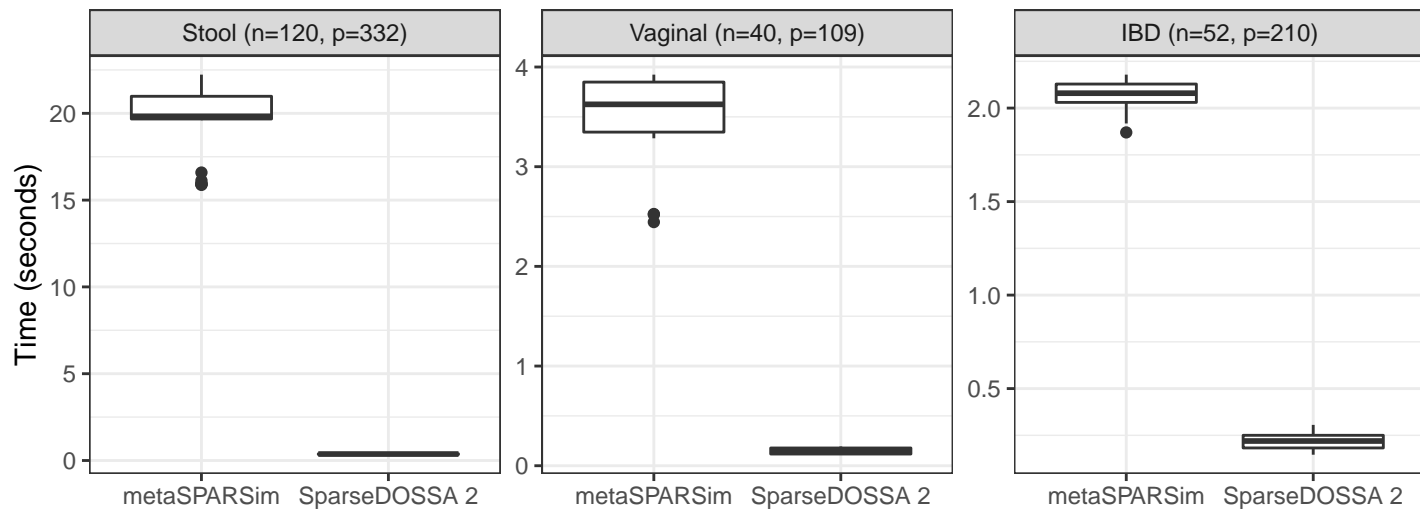

## B SparseDOSSA 2 process time costs

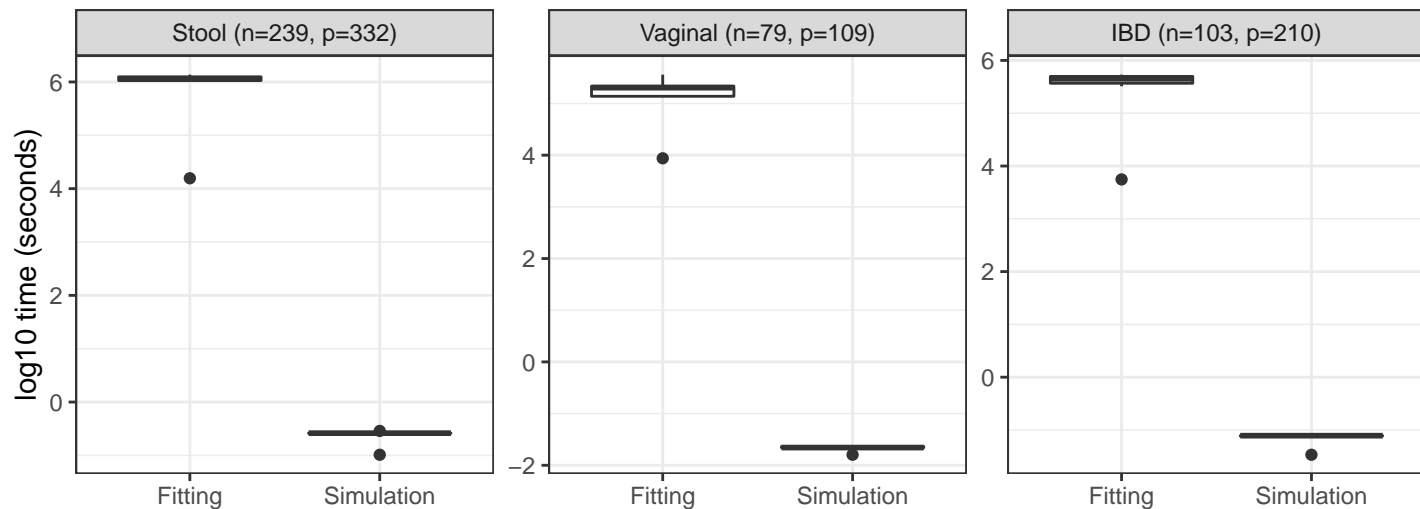

Supplement: S1 Fig — All computation evaluated as run on single Intel "Cascade Lake" cores. A) SparseDOSSA 2 simulation is faster than metaSPARSim across the evaluated real-world datasets. Results were aggregated across the 25 simulation evaluations (5 original dataset partitionings × 5 simulations) for each dataset. Note that here the simulation sample size n are halved compared to the actual sample size per dataset because only half the samples were simulated according to the training-testing partitioning paradigm (Methods). B) SparseDOSSA 2 fitting requires significant computation costs up front compared to its simulation process, analogous to sequence search database indexing. These are evaluated across the grid of tuning parameter λs per real-world dataset (fitting and simulation performed for full datasets). We note that the fitting costs can be potentially alleviated by 1) the algorithm is parallelizable and implemented as such, and 2) for a target template the fitting needs to be performed only once, whereas numerous simulation repetitions are required for e.g. benchmarking or power analysis. (PDF) [file pcbi.1008913.s001.pdf]

## Stool

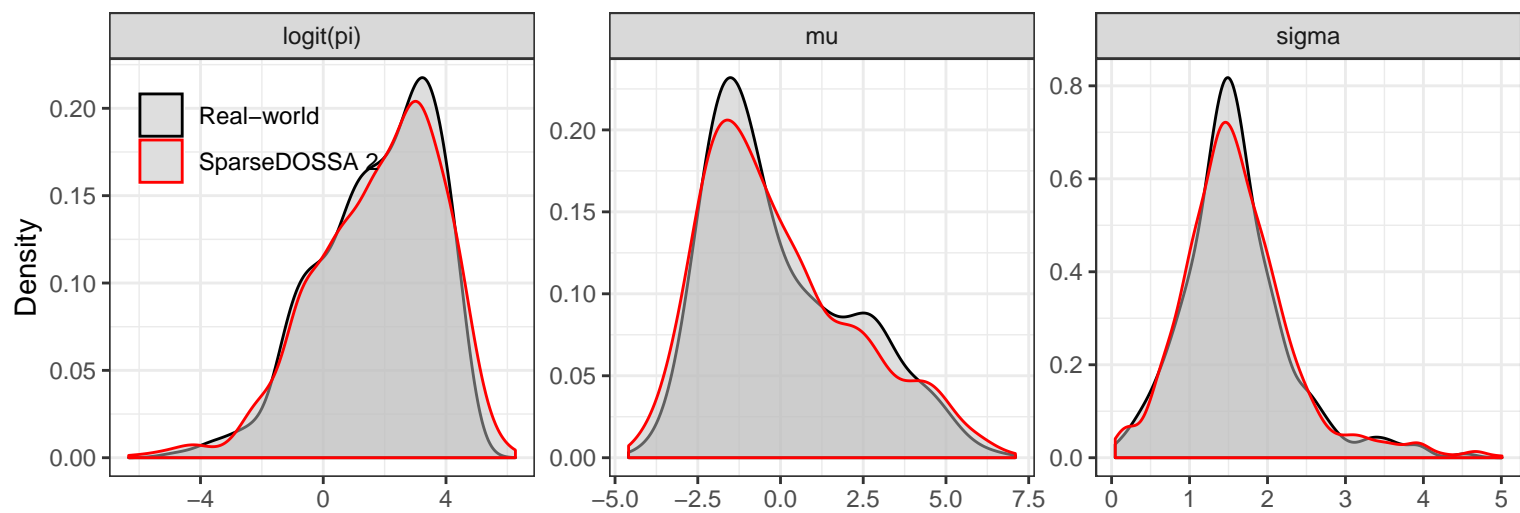

## Vaginal

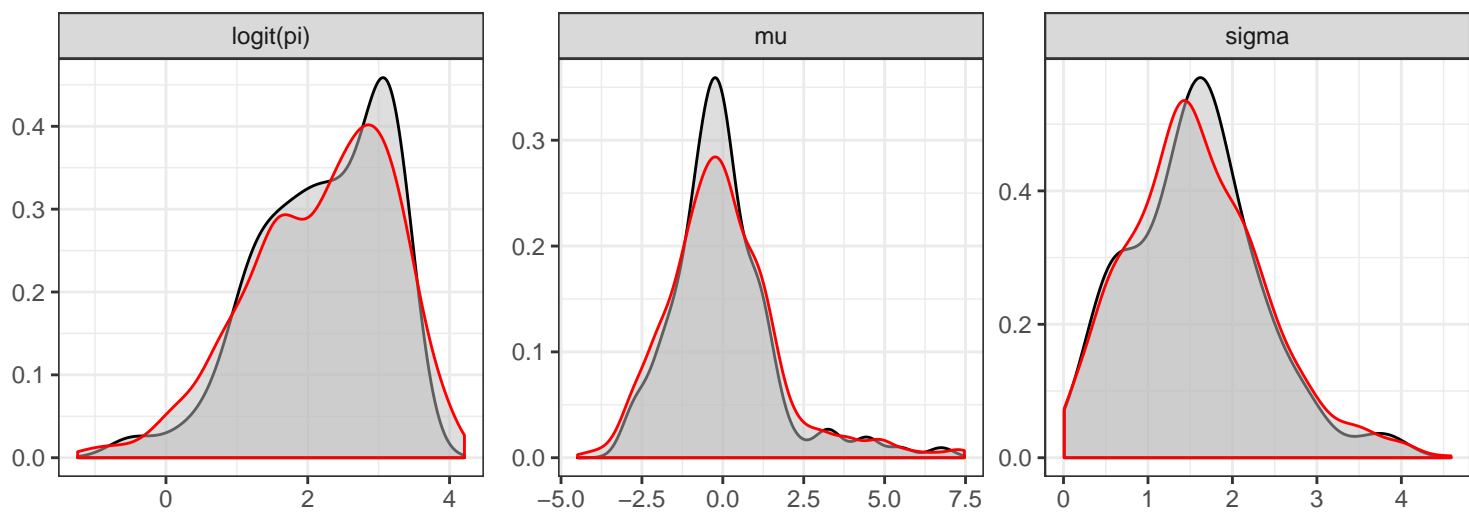

## IBD

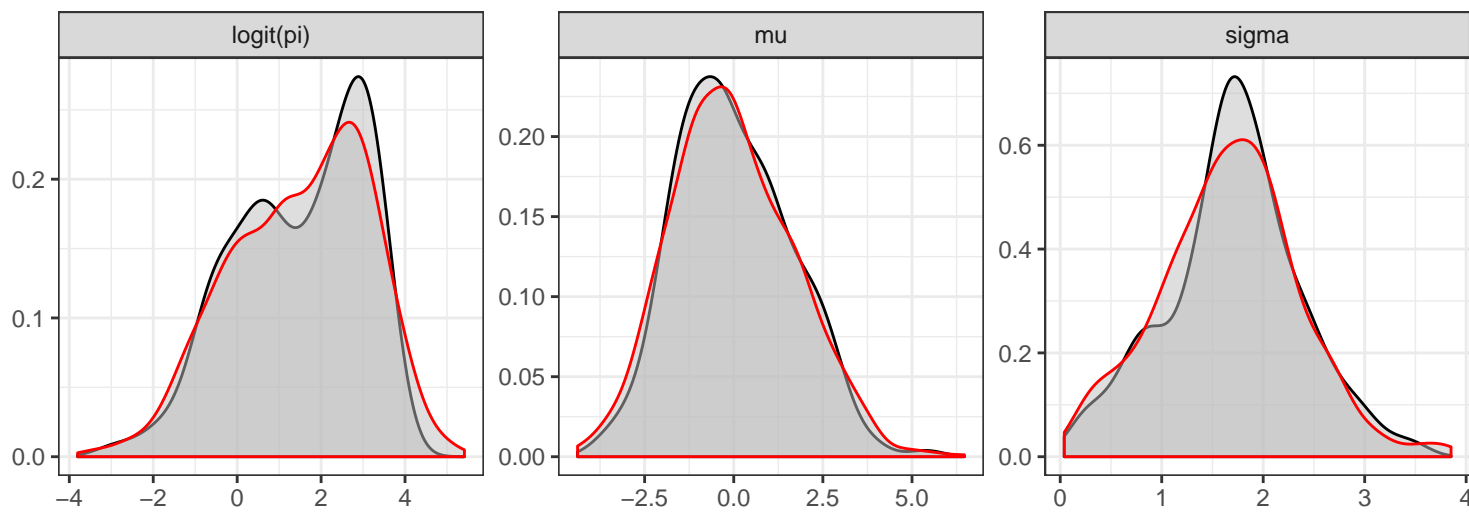

Supplement: S2 Fig — In each case, “new” microbial features were simulated with SparseDOSA 2’s three-dimensional Gaussian kernels, and compared with the original features by examining the distribution of their absence probability πj (logit transformed), mean log non-zero abundance μj, and standard deviation of log non-zero abundance σj. (PDF) [file pcbi.1008913.s002.pdf]

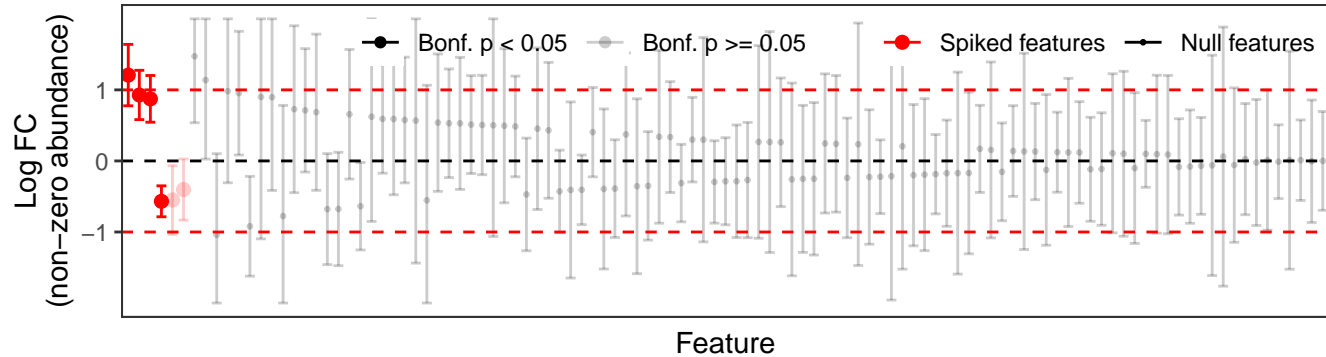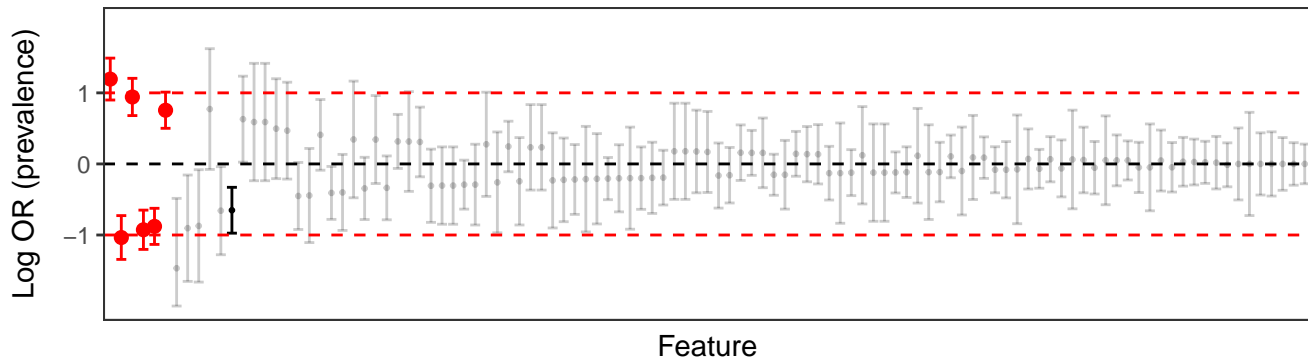

Supplement: S3 Fig — (PDF) [file pcbi.1008913.s003.pdf]

**A**

Simulated Stool Spearman Correlation

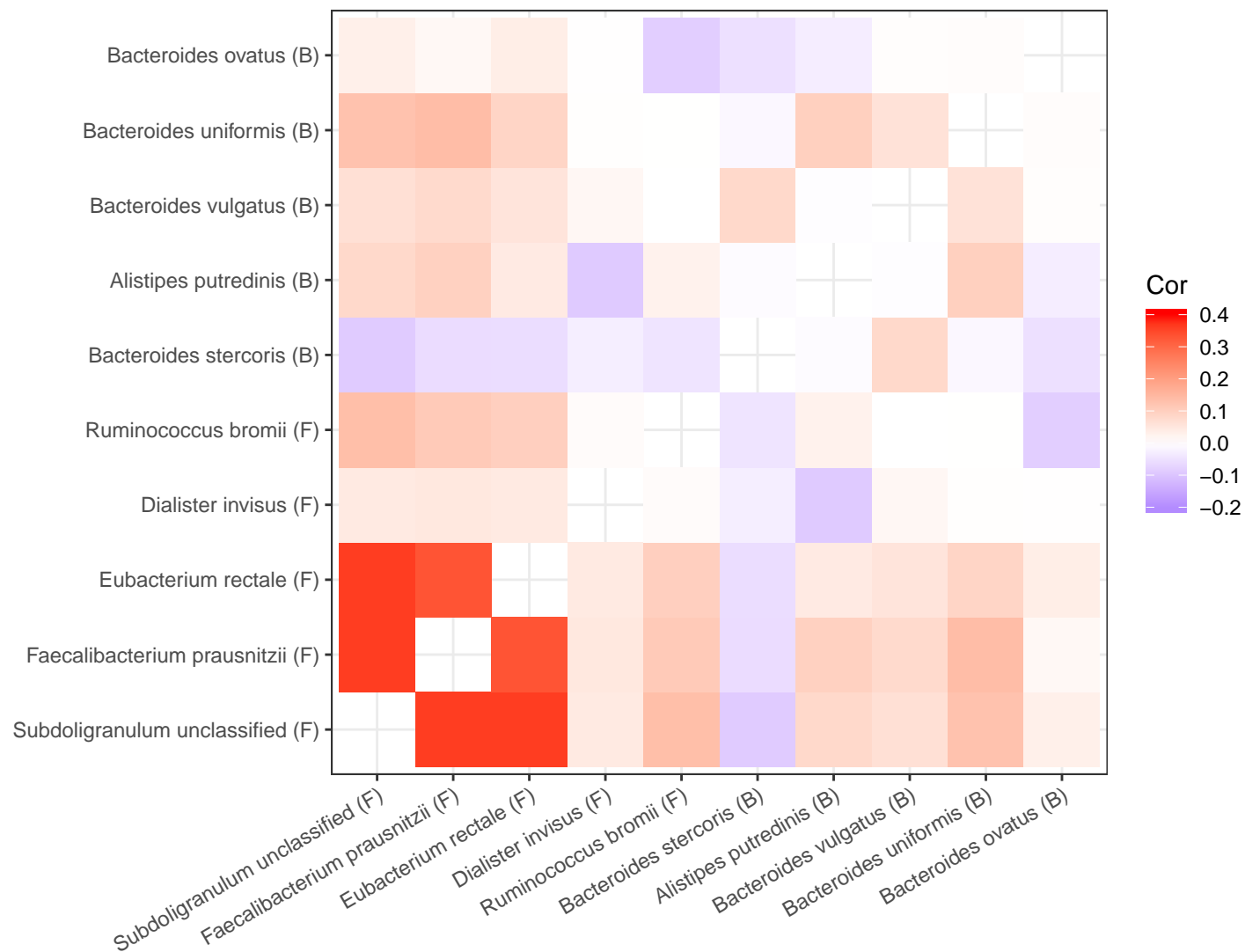**B**

Simulated Vaginal Spearman Correlation

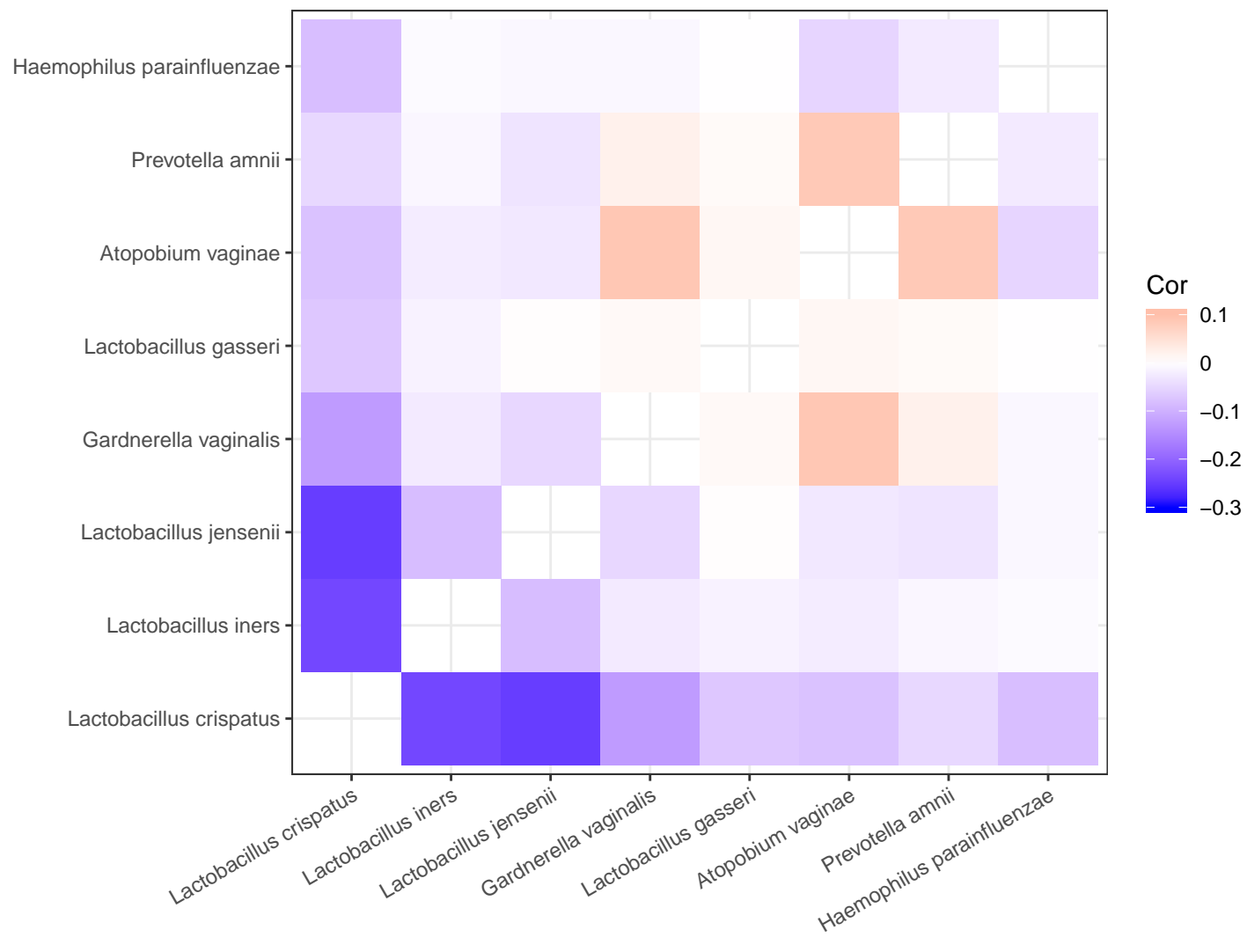

Supplement: S5 Fig — We simulated 100,000 microbial abundance profiles each based on the SparseDOSSA Stool and Vaginal models and examined the resulting simulated feature-feature Spearman correlations. Note that Spearman correlation does not differentiate between feature-feature correlations induced by biological interaction and compositionality; it is used purely to characterize co-variation patterns in the simulated microbial profiles, as opposed to making biological claims as to whether they are biological vs. technical. A) Simulated Spearman correlations in the top five most abundant Firmicutes-derived features (bottom left, abundance increasing top to bottom), and the top five most abundant Bacteroidetes-derived features (top right, abundance increasing bottom to top) from the Stool SparseDOSSA model. Strong positive correlations were observed among the Firmicutes species, whereas negative ones can often be observed between Firmicutes and Bacteroidetes species, as expected [9]. B) Simulated Spearman correlations in the top eight most prevalent and abundant species’ models from the Vaginal SparseDOSSA fit (bottom to top, left to right decreasing in average abundance). We observe strong co-exclusions between dominant Lactobacillus features, and positive correlations among a few less dominant species indicative of joint presence in the absence of Lactobacilli. (PDF) [file pcbi.1008913.s005.pdf]
