# Supplementary material for: A statistical model for describing and simulating microbial community profiles
Source: PLoS Comput Biol. 2021 Sep 13;17(9):e1008913. doi: 10.1371/journal.pcbi.1008913 (PMC8491899; doi:10.1371/journal.pcbi.1008913)
Supplement: S4 Fig — Top row: stool community results; bottom row: vaginal community results. (PDF) [file pcbi.1008913.s004.pdf]

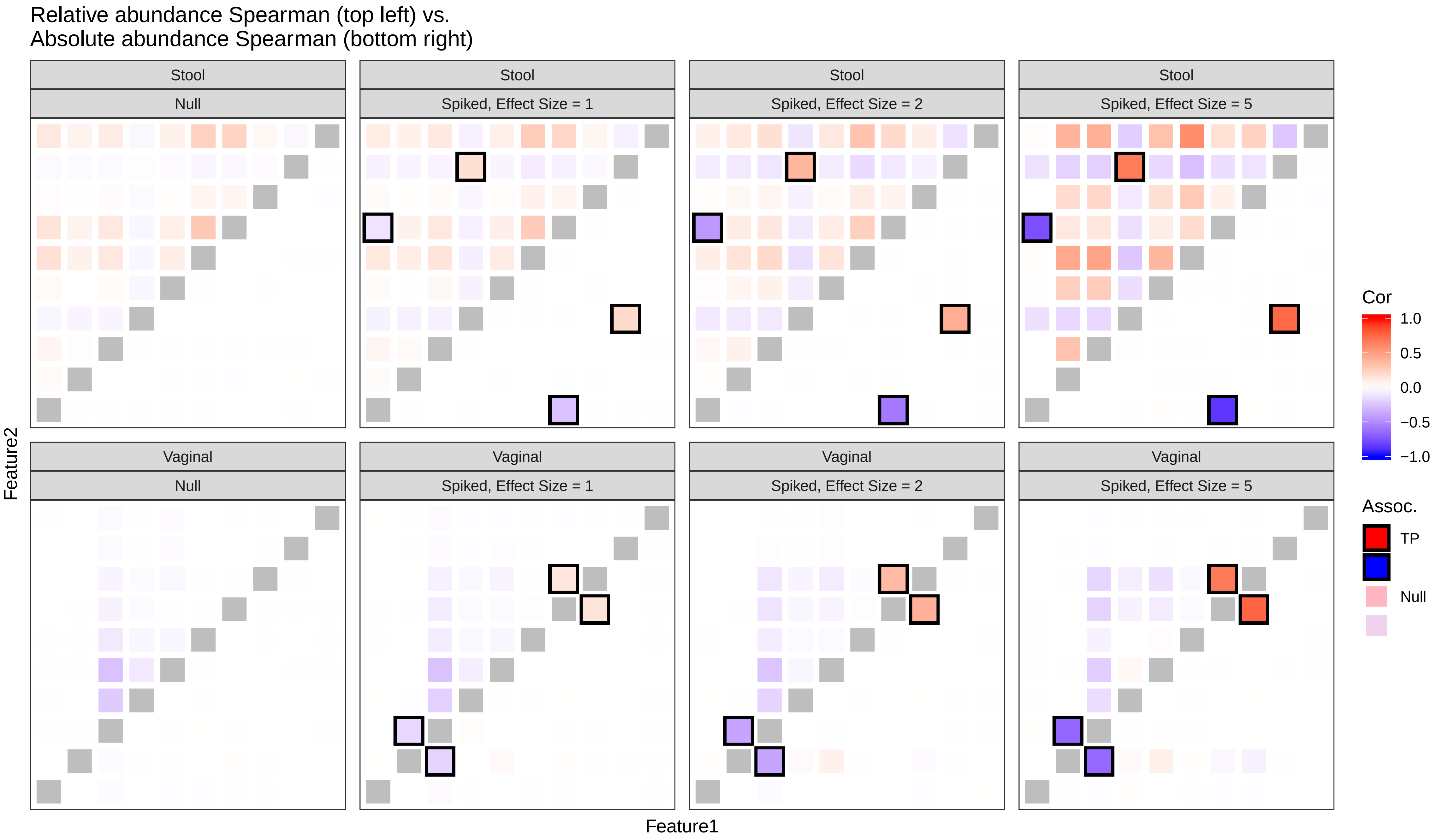

Relative abundance Spearman (top left) vs.
Absolute abundance Spearman (bottom right)
Stool
Null
Stool
Spiked, Effect Size = 1
Stool
Spiked, Effect Size = 2
Stool
Spiked, Effect Size = 5
Vaginal
Null
Vaginal
Spiked, Effect Size = 1
Vaginal
Spiked, Effect Size = 2
Vaginal
Spiked, Effect Size = 5
Feature2
Feature1
Cor
1.0
0.5
0.0
−0.5
−1.0
Assoc.
TP
Null
